# A generalized framework for *in vivo* detection of dopamine release using positron emission tomography

**DOI:** 10.1177/0271678X251362958

**Published:** 2025-09-19

**Authors:** Jordan U Hanania, Connor WJ Bevington, Ju-Chieh (Kevin) Cheng, Dongning Su, Alexandra Pavel, A. Jon Stoessl, Vesna Sossi

**Affiliations:** 1 Department of Physics and Astronomy, University of British Columbia, V6T 1Z1, Canada; 2 Pacific Parkinson’s Research Centre, University of British Columbia, V6T 2B5, Canada; 3 Center for Movement Disorders, Department of Neurology, 105738 Beijing Tiantan Hospital, Capital Medical University, Beijing, 100070, China; 4 Faculty of Medicine, Division of Neurology, University of British Columbia, V6T 2B5, Canada

**Keywords:** Dopamine, lp-ntPET, neurotransmitter release detection, PET imaging, RSD

## Abstract

Voxel-level detection of task-induced striatal dopamine (DA) release in humans is achievable with dynamic PET imaging, enabling complex studies of motor, cognitive, and reward tasks. We previously introduced a data-driven methodology termed Residual Space Detection (RSD), which improved detection of low-amplitude DA release, however its applicability was limited to detection of low-amplitude and/or localized effects. Here, we generalize RSD to broader DA release scenarios by introducing a novel model-based baseline time-activity curve prediction method in combination with non-local-means clustering (RSD-Hybrid-IMRTM). In simulations, RSD-Hybrid-IMRTM outperforms our previous methodology for detecting global striatal DA release, improving absolute detection sensitivity by 18% at 5% false positive rate, while also demonstrating the ability to track the magnitude of task-induced changes in synaptic DA concentrations in a noise-robust manner. As a proof of principle, we apply RSD-Hybrid-IMRTM to healthy controls and Parkinson’s disease subjects undergoing finger and foot tapping tasks. Results reveal expected group differences in parametric maps, parameter magnitudes, and functional segregation, demonstrating RSD-Hybrid-IMRTM’s utility for investigating neurotransmission in human cohorts.

## Introduction

Dopamine (DA) plays a major role in many aspects of brain function, including motor, cognitive, and reward processing.^1–3^ Dopaminergic dysfunction is involved in various brain disorders, most notably Parkinson’s disease (PD), where motor symptoms mainly reflect DA deficiency,^2^ as well as schizophrenia, where it is hypothesized that psychosis is related to excessive DA signaling.^4^ It is thus highly desirable to study DA behavior under a variety of physiological and pharmacological interventions to better understand DA processing in relation to task and disease.

Task-induced increases in synaptic DA concentrations above baseline levels (subsequently termed “DA release”) can be detected in select areas of the human brain by use of dynamic [^11^C]raclopride (RAC – a D2/3 receptor antagonist) PET imaging.^5–10^ Although this has traditionally been accomplished with two separate PET scans involving bolus injections of RAC, the first of which has the subject at rest to determine a baseline state while the second scan involves a sustained stimulus aimed to invoke DA release,^11^ a more recent approach for inducing and subsequently observing transient DA release involves a single 70+ minute bolus or bolus+infusion PET scan.^7^ Under this protocol a baseline state is maintained through the first 30–40 minutes of scanning, after which the subject performs a task designed to induce DA release, such as smoking,^8,9,12^ engaging in a motor activity,^13^ or reward-based learning.^14^ This causes a transient voxel-level perturbation to the dynamic PET data as a result of binding competition between DA and RAC at receptor sites: the increased synaptic DA concentration limits subsequent RAC binding, resulting in a localized time-varying decrease in the tracer time activity curve (TAC). As DA concentrations cannot be directly measured *in vivo* in humans, current detection methods rely on semi-quantitative proxy measures derived from RAC TACs, which are deemed proportional to *relative* DA concentration changes.

There are two major components of a DA release detection framework: (1) modeling of ‘baseline’ behavior, and (2) task-related DA release modeling. The first relates to a measured voxel TAC’s expected behavior in the absence of DA release and is primarily achieved by analysis of pre-task TAC data. The majority of developments in the present work are aimed at improving the accuracy of the baseline definition, as this step is crucial for reliable predictions of the second component. Task-related DA release modeling may take a variety of forms, including binary or probabilistic detection, prediction of a semi-quantitative metric related to DA release magnitude, or estimation of DA release timecourses.

We recently proposed a data-driven framework, termed Residual Space Detection (RSD),^15^ that was found to outperform the more commonly used method, linear parametric neurotransmitter PET (lp-ntPET), in a situation of localized low-amplitude dopamine release. In particular, the enhanced performance of RSD in such a DA release scenario was due to its ability to better define baseline TAC behavior which led to higher detection sensitivity.

While RSD in its original implementation extended the ability of lp-ntPET to detect localized and low-magnitude DA release, it also had limitations (outlined in Table 1) which led to its suboptimal performance in a more general DA release scenario, where it was consistently outperformed by lp-ntPET. The primary limitation was the assumption of DA release being localized to small striatal sub-regions: as RSD relied on finding voxel TACs within the striatum that were free of DA release signal to use as regressors, if release was of a large magnitude and/or spatial extent, RSD’s defined baseline TACs would be biased (see Theory).

**Table 1.** Limitations of the previously published RSD methodology and improvements made in this work.

Limitation of previous RSD implementation (RSD-k-means)^15,16^	Solution (RSD-Hybrid-IMRTM)
1. **Coarse graining.** k-means was used to identify three regions with similar pre-task voxel TACs, with baseline regressors defined as the mean of these voxel TACs. These regressors were unable to span the full range of voxel TACs, resulting in biased baseline TAC predictions for boundary voxels within each k-region.	Non-local means (equations (5) and (6)) replaces k-means, providing finer sampling of the data to determine similar pre-task behavior.
2. **Localized release assumption.** RSD-k-means assumed localized release such that the baseline regressors—defined by averages of voxels TACs—were minimally biased by voxels with release. This feature was appropriate if DA release was indeed localized, however in the case of more wide-spread DA release RSD-k-means was only capable of detecting regions of stronger-than-average DA release amplitude.	Iterative MRTM (IMRTM), developed below and in more detail in the Supplemental Material, situationally provides model-derived baseline TACs to RSD, enabling detection and characterization of spatially extended and/or high-amplitude DA release.
3. **Iteration.** An iterative step to remove voxels likely containing DA release from the definition of baseline regressors was necessary to improve accuracy of baseline predictions, but there was some arbitrariness in determining the removal threshold.	lp-ntPET provides a non-arbitrary removal/replacement decision for voxel TACs that likely contain DA release, by use of an F-test.

The aims of this work are: (1) to generalize the RSD methodology to be applicable to a variety of tracer binding conditions (e.g. spatially heterogeneous receptor densities or low basal DA levels present in PD)^17,18^ and DA release scenarios (e.g. extended vs local release, low- vs high-amplitude), (2) demonstrate robustness of RSD to track DA release magnitude in the presence of noise, and (3) present a proof-of-principle application of RSD to human cohorts. The goal is thus to demonstrate that the generalized method has a similar performance to lp-ntPET in a scenario of diffuse, relatively high DA release, while still maintaining its high detection sensitivity for low level localized DA release.

The paper proceeds as follows. After providing a succinct background on lp-ntPET and the original implementation of RSD, a novel baseline TAC prediction method is developed as an iterative extension to the multilinear reference tissue model (Iterative MRTM; IMRTM).^19^ Then a generalized RSD framework is developed, termed RSD-Hybrid-IMRTM; “hybrid” referring to the combination of both data-driven and kinetic model-based baseline TAC predictions to achieve more robust voxel baseline TACs and residuals. A residual form of lp-ntPET making use of the improved baseline TAC predictions of RSD-Hybrid-IMRTM is then introduced to provide more robust estimates of semi-quantitative metrics that track DA release magnitude ( γ of equation (1) and time-varying $BP_{ND}$ of equation (16)). The detection performance of RSD-Hybrid-IMRTM is then compared to our previous version of RSD and the standard lp-ntPET detection framework. Next, the capabilities of the semi-quantitative DA release metrics in each framework— γ in lp-ntPET (equation (1)) and β in RSD (equation (11))—are explored using simulations. When applied to human scans from HC and PD subjects performing finger and foot tapping tasks (part of a larger study ongoing in our center), we show that RSD provides more meaningful group differences in analyses of T-tests, parameter distributions, and functional segregation. Finally, practical considerations and limitations are discussed regarding interpretation of DA release metrics across subject groups.

## Theory

### Existing DA release detection frameworks

#### Lp-ntPET

The linear parametric neurotransmitter PET model (lp-ntPET; equation (1)) uses a library of pre-defined release timecourses to simultaneously predict baseline and DA release behavior on a voxel level:^20^

(1) $$C_{T}\left(t\right)=R_{1}C_{R}\left(t\right)+k_{2}\int_{0}^{t}C_{R}\left(u\right)\text{d}u-k_{2a}\int_{0}^{t}C_{T}\left(u\right)\text{d}u-\gamma \int_{0}^{t}C_{T}\left(u\right)h\left(u\right)\text{d}u$$

where $C_{T}(t)\;$ is the target region (i.e. voxel) tracer concentration, $C_{R}(t)$ is the reference region tracer concentration, and the temporal shape of DA release is modeled by

(2) $$h\left(t\right)=\theta \left(t-t_{D}\right){\left(\frac{t-t_{D}}{t_{P}-t_{D}}\right)}^{\alpha }\text{exp}\left[-\alpha \left(\frac{t-t_{P}}{t_{P}-t_{D}}\right)\right]$$

which relates to the DA-induced TAC perturbation. Here the parameters ( $t_{D}:$ start time, $t_{P}$ : peak time, α : sharpness) are discretized according to *a priori* estimates of possible shapes, while the baseline parameters ( $R_{1},\;k_{2},\;k_{2a}$ ) as well as γ —a proxy parameter for DA release magnitude—are solved by weighted least squares minimization. The standard detection protocol separately fits the multilinear reference tissue model (MRTM; first three terms of equation (1)) and lp-ntPET to striatal voxel TACs; voxels that are fit best by lp-ntPET exhibit statistically significant F-statistics and are thus deemed likely to contain DA release.^7,8^

#### RSD

Residual Space Detection (RSD) is a method that aims to predict what voxel TACs would be in a baseline state (i.e. if no DA release occurred) and compares predicted baseline TACs to measured voxel TACs by extracting residual behavior via a percentage difference. Applying a General Linear Model to voxel residuals provides estimates of “percent signal change from baseline”, used as a proxy for DA release.^15^

The major innovation of RSD compared to lp-ntPET was to perform baseline TAC predictions and DA release metric predictions in separate steps, which helped to improve robustness of (1) determination of baseline parameters, and (2) estimation of parameters relating to release magnitude. Specifically relating to (1), the lp-ntPET baseline parameters ( $R_{1},\;k_{2},\;k_{2a}$ ) may take on unrealistic values due to the numerous degrees of freedom in the model, in conjunction with the fact that lp-ntPET does not use information from multiple voxels to constrain single-voxel parameter estimates in the standard detection framework. Another issue for baseline estimation is that if DA release is present, the $k_{2a}\int_{0}^{t}C_{T}\left(u\right)du$ term (equation (1)) is ‘contaminated’ by release-induced signal in $C_{T}(t)$ , potentially leading to inaccurate fitting parameters. RSD addressed these issues by fitting a set of 3 baseline TAC regressors to each voxel, in effect limiting the parameter space of voxel ( $R_{1},\;k_{2},\;k_{2a}$ ) values and reducing false positives.

Regarding issue (2), the lp-ntPET parameter γ is dependent on the choice of $h\left(t\right)$ (equation (2)), for which similar model TAC fits can result in drastically different γ estimates (Supplemental Figure 1). RSD simplified DA-related parameter estimation by first extracting “residuals” relating to DA-induced perturbations within voxel TACs, defined as the percent difference between a predicted baseline TAC and the measured voxel TAC. Residuals were then fit to a single TAC-level predictor function P(t) (equations (11) and (12)), rather than several kinetic-level h(t) functions, which improved robustness of parameter estimates.

### Baseline TAC prediction

An important aspect of RSD is the prediction of baseline TACs for each voxel in the region of interest (typically the striatum), which can then be used to determine the magnitude of task-related deviations in the measured voxel TACs as a proxy for DA release. Possible approaches for obtaining voxel-level baseline TAC predictions include (i) using the measured data, (ii) using a model, or (iii) using a hybrid of model- and data-driven TACs.

#### Data-driven baseline TAC prediction and its limitations (RSD-k-means)

Our previously published work used the measured data alone to construct baseline TACs. In brief, our previous method (hereafter: RSD-k-means)^15^ used k-means clustering on pre-task voxel TACs to identify three regions of the striatum, each containing voxels with similar pre-task baseline tracer binding characteristics. The value of k = 3 was chosen to obtain baseline regressor TACs encapsulating a range of binding potential ( $BP_{ND}$ ) values of the data, providing a TAC for relatively high-, mid-, and low- $BP_{ND}$ . Computing the mean TAC for each k-region provided a set of three baseline TACs with different binding characteristics, which could then be fit to each voxel TAC by multiple regression, resulting in a voxel-specific baseline TAC. RSD-k-means provided reasonable baseline TAC predictions for the majority of voxels in simulations of low-amplitude, localized DA release in the presence of heterogeneous RAC binding, ultimately increasing detection sensitivity compared to the model-based lp-ntPET method. This approach was inherently limited to detecting localized release (Table 1), since it relied on the majority of voxels to be in a baseline state in order to derive its data-driven baseline TAC predictions.

#### Model-based baseline TAC prediction (Iterative MRTM)

To ameliorate the issues of data-driven detection, we now introduce a novel model-based method called Iterative MRTM (IMRTM), used for extracting baseline TAC behavior from a measured voxel TAC which may contain task-induced neurotransmitter release. This method first fits the traditional MRTM model prior to the task start, $t_{D}$ , and then extrapolates the fit to all time points:

(3) $$\begin{array}{c} \mathrm{Fit}:C_{T}\left(t\right)=R_{1,\mathrm{pre}}C_{R}\left(t\right)+k_{2,\mathrm{pre}}\int_{0}^{t_{D}}C_{R}\left(u\right)\text{d}u\\ -k_{2a,\mathrm{pre}}\int_{0}^{t_{D}}C_{T}\left(u\right)\text{d}u;t<t_{D}\\ {\to C}_{\mathrm{IMRTM}}^{\left(1\right)}\left(t\right)=R_{1,\mathrm{pre}}C_{R}\left(t\right)\\ +k_{2,\mathrm{pre}}\int_{0}^{t}C_{R}\left(u\right)\text{d}u-k_{2a,\mathrm{pre}}\int_{0}^{t}C_{T}\left(u\right)\text{d}u \end{array}$$

**Figure 1.**
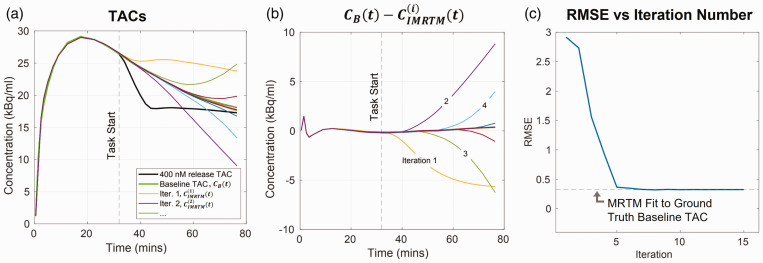
Demonstration of IMRTM baseline convergence for noise free release TAC $C_{T}(t)$ . (a) Ground truth release (black) and baseline (green) TACs, with iterative MRTM baseline TAC predictions $C_{\mathrm{IMRTM}}^{\left(i\right)}(t)$ . (b) Absolute residuals ${C_{B}\left(t\right)-C}_{\mathrm{IMRTM}}^{\left(i\right)}(t)$ displaying temporal convergence of IMRTM to the true baseline and (c) Root mean square error (RMSE) of $C_{\mathrm{IMRTM}}^{\left(i\right)}(t)$ to ground truth baseline TAC $C_{B}\left(t\right)$ , vs iteration number.

This provides a first approximation to a voxel-specific baseline TAC, $C_{\mathrm{IMRTM}}^{\left(1\right)}(t)$ , with iteration number denoted in the superscript. However, in the presence of DA release, $C_{T}(t>t_{D})$ contains a task-related dip, resulting in a positively biased $C_{\mathrm{IMRTM}}^{\left(1\right)}(t>t_{D})$ (Figure 1). To resolve this, $C_{\mathrm{IMRTM}}^{\left(1\right)}(t)$ is substituted back into equation (3) in place of $C_{T}\left(t\right)$ on the right-hand side of the equation and this procedure is iteratively repeated. Defining $C_{\mathrm{IMRTM}}^{\left(0\right)}\left(t\right)=C_{T}\left(t\right)$ , the $n^{th}$ IMRTM baseline TAC estimate is

(4) $$C_{\mathrm{IMRTM}}^{\left(n\right)}(t)=R_{1,\mathrm{pre}}C_{R}\left(t\right)+k_{2,\mathrm{pre}}\int_{0}^{t}C_{R}\left(u\right)\text{d}u-k_{2a,\mathrm{pre}}\int_{0}^{t}C_{\mathrm{IMRTM}}^{\left(n-1\right)}(u)\text{d}u$$

which results in stable baseline TAC predictions after around 10 iterations, at which point fluctuations in root mean squared error relative to the ground truth baseline TAC fall below 1% (Figure 1(c)).

To demonstrate the IMRTM procedure, we used the ntPET^21^ model to simulate a noise-free [^11^C]raclopride TAC, $C_{T}\left(t\right)$ , containing a high peak amplitude of DA release (400 nm above baseline; detailed simulation parameters are given in Supplemental Table 1). This DA release TAC and its theoretical baseline TAC are shown in Figure 1(a) (black and green TACs, respectively). The DA release-containing TAC, $C_{T}\left(t\right)$ , was subject to IMRTM (equation (3) and (4)), producing subsequent baseline TAC predictions that eventually converge to the MRTM fit of the theoretical baseline TAC (Figure 1(b) and (c)). Note that IMRTM is ultimately limited to recovering the MRTM fit to the ground truth baseline TAC, since it still relies on a simplified one-compartment tissue model to fit to data simulated using two-compartment kinetics. For mathematical justification of the IMRTM procedure, see the Supplemental Material section “*Convergence of IMRTM*”.

#### Hybrid baseline TAC prediction (RSD-Hybrid-IMRTM)

While IMRTM provides the ability to predict baseline TACs using a model, it relies on a good fit to the measured voxel TAC prior to the task, which may fail due to pre-task noise or simply because the pre-task data does not capture the voxel’s true kinetics.^22^ Therefore, we aggregate both the IMRTM-derived baseline TACs and the measured data into a framework referred to as RSD-Hybrid-IMRTM, to achieve more robust voxel-level baseline TAC predictions. This removes RSD-k-means’ “model-free” requirement, instead opting to use a hybrid approach making use of both model- and data-derived baseline TACs.

The first step of the proposed method is to find voxels with similar kinetics, so that data from other similar voxels may be combined to reduce noise. To do this, we apply MRTM on pre-task data of voxel TACs inside a striatal mask, extracting voxelwise striatal MRTM parameters ( $R_{1},k_{2},k_{2a}$ ). As the magnitudes of these parameters are different, we z-transform each parameter across striatal voxels, $\left({\tilde{R}}_{1},{\tilde{k}}_{2},{\tilde{k}}_{2a}\right)=\left(z\left(R_{1}\right),z\left(k_{2}\right),\;z\left(k_{2a}\right)\right)$ , effectively removing unit/scale differences of the parameters and enabling clustering methods using Euclidean distance.

An inherent limitation of our previous method (Table 1) was that $\left({\tilde{R}}_{1},{\tilde{k}}_{2},{\tilde{k}}_{2a}\right)$ values were clustered on a coarse-grained level; k-means was used to identify three regions with similar pre-task voxel TACs, with baseline regressors defined as the spatial mean of these voxel TACs. Coarse graining refers to the inability of these regressors to span the full range of voxel TACs, resulting in biased baseline TAC predictions for boundary voxels within each k-region. To avoid coarse graining when defining voxel baseline TACs we now implement non-local means (NLM) in this space, where each voxel j is assigned a set of Gaussian weights based on its distance to all other voxels i :

(5) $$w_{i,j}=\exp\left(-\frac{{\left|{\vec{d}}_{j}-{\vec{d}}_{i}\right|}^{2}}{h^{2}}\right);\;w_{j,j}=0$$

where ${\vec{d}}_{n}=({\tilde{R}}_{1,n},{\tilde{k}}_{2,n},{\tilde{k}}_{2a,n})$ is the position of voxel n in the z-scored parameter space and $h^{2}$ is a hyperparameter determining the weight of voxels as a function of “distance” (set to $h^{2}=0.5$ ; see Supplemental Figure 2).

As RSD-Hybrid-IMRTM aims to predict baseline TAC behavior by aggregating measured data (some of which is expected to contain DA release), it needs an objective criterion for avoiding use of release-contaminated signal in its baseline predictions. This objective criterion is provided by a pass of lp-ntPET fitting; after obtaining voxel-level F-statistics with lp-ntPET, F , any voxel i with $p\left(F_{i}\right)<0.05$ (i.e. likely DA release-containing) has its TAC replaced with an IMRTM model-derived TAC in the NLM weighted average (equation (7)). While $p\left(F\right)<0.05$ will likely not remove all contaminant signal, it appears to be a sufficient threshold to remove a significant amount of the highest-release signal and thus provide clean baseline TACs.

A baseline regressor set is thus constructed using IMRTM-derived TACs for voxels that were deemed significant under lp-ntPET fitting, along with measured voxel TACs that were not deemed significant:

(6) C˜it=Cit; pFi≥0.05CIMRTM,it; pFi<0.05

Finally, voxelwise baseline TAC predictions are made using the weights defined by NLM (equation (5)) by first preferentially averaging across model- and data-derived baseline TACs with similar kinetics:

(7) $$C_{\mathrm{NLM},j}\left(t\right)=\frac{\sum_{i}w_{i,j}{\tilde{C}}_{i}\left(t\right)}{\sum_{i}w_{i,j}}$$

Then the final baseline TAC prediction ${\hat{C}}_{j}$ is calculated by scaling $C_{\mathrm{NLM},j}$ to the measured voxel TAC $C_{j}$ and correcting for spill-in from surrounding tissue. This is achieved by first determining $\theta_{\mathrm{NLM}}$ and $\theta_{R}$ by non-negative least squares fitting to the measured pre-task voxel TAC,

(8) $$C_{j}\left(t\right)=\theta_{\mathrm{NLM}}C_{\mathrm{NLM},j}\left(t\right)+\theta_{R}C_{R}\left(t\right);\;t<t_{D};\;\theta_{\mathrm{NLM}},\theta_{R}>0$$

then extrapolating the fit to all time points:

(9) $${\hat{C}}_{j}\left(t\right)=\theta_{\mathrm{NLM}}C_{\mathrm{NLM},j}\left(t\right)+\theta_{R}C_{R}\left(t\right);\;\forall t$$

Equation (8) assumes that voxel values are a linear combination of true signal and spill-in from surrounding voxels devoid of specific binding, thus the non-negativity constraint enforces this physiological assumption. In practice, $\theta_{R}$ is only non-zero at edge voxels and is typically of low magnitude.^15^ Its inclusion acts to reduce false positives caused by the presence of non-specific signal which could appear as a post-task TAC decrease relative to baseline.

RSD then proceeds to take the percent difference of the measured voxel TAC $C_{j}\left(t\right)$ from its predicted baseline TAC ${\hat{C}}_{j}(t)$ , to define percent-difference residuals $R_{\mathrm{pct},j}(t)$ as

(10) $$R_{\mathrm{pct},j}(t)=100\%\times [1-C_{j}\left(t\right)/{\hat{C}}_{j}(t)]$$

Defining residuals in this way, rather than as a subtraction, results in residual magnitudes invariant to task start timing and insensitive to $BP_{ND}$ (Supplemental Figure 3). Residuals are then analyzed using a General Linear Model (GLM):

(11) $$R_{\mathrm{pct}}\left(t\right)=\beta P\left(t\right)+\epsilon \left(t\right);\;\max\left(P\left(t\right)\right)=1$$

P(t) is an assumed residual TAC response to DA release, modeled as a single gamma variate function convolved with a task-block vector,^15^ ϵ(t) is the error in the fit, and β is the fit parameter, interpretable as a voxel TAC’s maximal percent decrease from baseline. More specifically, $P\left(t\right)$ is given by

(12) $$P\left(t\right)=\lambda \left(\theta \left(t-t_{D}\right)-\theta \left(t-t_{F}\right)\right)*\left(\frac{t}{L}\text{exp}\left(-\frac{t}{L}\right)\right);\underset{\lambda }{\max}\left(P\left(t\right)\right)=1$$

where θ is the Heaviside function, $t_{D}$ is the delay time indicating the start of the task relative to the scan start time, $t_{F}$ is the task end time ( $t_{F}>t_{D}$ ), λ is a normalization constant, and L is a parameter governing the broadness of the gamma variate response function. Empirically, we found that using a gamma variate function with L=10  min gave P(t) that reasonably matched the shape of residuals derived from noiseless simulations. This single predictor function $P\left(t\right)$ is unrelated to the library of predictor functions h(t) employed by the standard lp-ntPET framework: P(t) is a TAC-level response describing the shape of predicted TAC deflection from baseline, while h(t) is a kinetic-level response describing the time-varying alteration of the $k_{2a}$ parameter as caused by DA release.

### Residual lp-ntPET

It is often desirable to determine measures relating to the time-varying binding potential,^23^ for which the benefits of RSD-Hybrid-IMRTM’s baseline prediction can be used to obtain more stable predictions of γ and/or h(t) (and thus $BP_{ND}\left(t\right)$ ; equation (16)), by defining a residual form of lp-ntPET. Using absolute residuals derived from the RSD predicted baseline TAC ${\hat{C}}_{j}(t)$ (equation (9)) and voxel TAC $C_{j}(t)$ ,

(13) $$R_{\mathrm{abs},j}\left(t\right)={\hat{C}}_{j}\left(t\right)-C_{j}\left(t\right)$$

then noting that ${\hat{C}}_{j}(t)$ is fit well by MRTM (as it is a baseline TAC) and assuming that $C_{j}(t)$ is fit well by lp-ntPET, along with the enforcement that lp-ntPET uses the same $R_{1}$ and $k_{2}$ values, the residual form of lp-ntPET is then

(14) $$R_{\mathrm{abs},j}\left(t\right)=\left(R_{1}C_{R}\left(t\right)+k_{2}\int_{0}^{t}C_{R}\left(u\right)\text{d}u-k_{2a}\int_{0}^{t}{\hat{C}}_{j}\left(u\right)\text{d}u\right)-(R_{1}C_{R}\left(t\right)+k_{2}\int_{0}^{t}C_{R}\left(u\right)\text{d}u-k_{2a}\int_{0}^{t}C_{j}\left(u\right)\text{d}u-\gamma \int_{0}^{t}C_{j}\left(u\right)h\left(u\right)\text{d}u)=\gamma \int_{0}^{t}C_{j}\left(u\right)h\left(u\right)\text{d}u-k_{2a}\int_{0}^{t}R_{\mathrm{abs},j}\left(u\right)\text{d}u.$$

This may be solved using linear regression similar to the typical lp-ntPET procedure. Note that the benefits of percent-difference residuals (equation (10)) cannot be utilized here, as absolute residuals are required to simplify terms in equation (14). We provide results from this model as applied to human subjects below, with example γ distributions and residual fits provided in Supplemental Figures 4 and 5.

## Methods

### Simulation design

Noiseless TACs were simulated following the ntPET model.^9^ Kinetic parameters derived from a healthy control subject scanned under baseline conditions were used alongside pre-specified synaptic DA concentration increases and timings to obtain simulated TACs (see Supplemental Table 1 and Supplemental Material section “*Simulation details*” for more information). It is noted that IHYPR4D^24^ post-processing was used for all noisy simulations and human scans for denoising (spatial kernel size of 7.2 mm isotropic, temporal kernel size of 2 frames, 1 iteration).

Two simulation sets were performed in this work to address Aims 1 and 2, both simulating a single 10-minute functional task block starting at 36 minutes:

#### Detection performance

**Figure 2.**
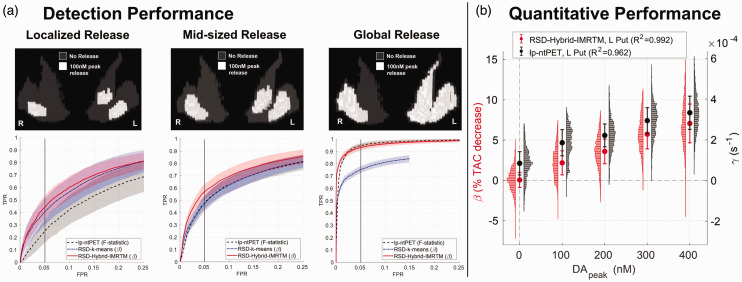
Simulation results. (a) Detection performance for low-amplitude DA release in localized, mid-sized, and global clusters (50 noisy realizations each), comparing the novel RSD-Hybrid-IMRTM to RSD-k-means and lp-ntPET. True positive rate (TPR) is calculated across all ground truth clusters, while false positive rate (FPR) is calculated across the release-free right caudate region. Note, the abscissa is limited to the range 0–25% FPR to highlight methodological differences and (b) quantitative tracking of DA release magnitude in the left putamen cluster (85 voxels) for RSD-Hybrid-IMRTM and lp-ntPET. Note the difference in scale of the y-axes.

Clusters of low-amplitude DA release (100 nM increase from a basal DA concentration of 96.4 nM) were placed in the left and right anterior putamen and left caudate. To compare the detection performance of the methods described in the previous section, 50 noisy realizations (NRs) were performed for each scenario of localized clusters (17–22% of ROI size), mid-sized clusters (24–48% of ROI size), and full-ROI clusters of DA release (Figure 2). lp-ntPET was applied with 17 basis functions to allow flexibility in detection ( $t_{D}=36\;\min,\;\alpha =1$ fixed, $t_{P}$ varied from 37–51 min ).

#### Quantitative performance

20 NRs each of 0, 100, 200, 300, and 400 nM DA release were simulated using the localized cluster set from simulation 1. The intent of these simulations was to examine the quantitative behavior of the methods and their ability to track DA release magnitude. lp-ntPET was applied with 1 optimal basis function ( $t_{D}=36\;\min$ , $t_{P}=42\;\min$ , α=1 ) for this dataset to minimize variance in γ .

### Simulation analysis

#### Detection performance

Striatal parametric maps output from RSD-Hybrid-IMRTM ( β values) and lp-ntPET (F-statistics) had in-plane edge voxels removed to minimize false positives caused by partial volume effects. Rather than determining detection performance by means of formal statistical tests on β and F , detection performance was assessed across varied thresholds of the raw parametric maps, ranging from 0 to the maximum parameter value across all NRs. This enabled empirical determinations of true positive rates (TPR) for simulated DA release clusters across all values of false positive rate (FPR).

Thus, for each NR, threshold value, and method, a binarized map was obtained, from which TPR was computed across all simulated DA release clusters and FPR was computed within the release-free right caudate. Averaging across NRs for each threshold’s TPR and FPR resulted in a receiver operating characteristic (ROC) curve for each method (Figure 2(a)).

#### Quantitative performance

β and γ values output from RSD-Hybrid-IMRTM and lp-ntPET, respectively, were used to track changes in synaptic DA levels within ground truth clusters across noisy realizations. For each small release cluster (L caudate: 46 voxels; L putamen: 85 voxels; R putamen: 69 voxels; voxel size: 1.4 × 1.4 × 2.8 mm) metric values were averaged across voxels and noisy realizations (Figure 2(b)). Histograms of parameter distributions for all clusters and baseline voxels are included in Supplemental Figure 6.

### Human scans

To examine the ability of RSD-Hybrid-IMRTM to find group differences in human subjects, we present results from HC and PD cohorts, with subjects undergoing separate finger tapping and foot tapping [^11^C]RAC scans. Differences in activation maps between HC and PD subjects were theoretically expected to include loss of task performance segregation and compensation for motor function deficits in PD by diffuse striatal regions.^25,26^

#### Scan protocol

Thirteen PD subjects (5 female; mean age = 66.2, SD = 7.9, range = 55–78) and fifteen healthy controls (8 female; mean age = 64.2, SD = 8.7, range = 47–77) were scanned on the GE SIGNA PET/MR scanner. All HC participants tapped with their dominant right hand/foot, while PD subjects tapped the limb on their most affected motor side (7 right, 6 left). At 36 minutes post bolus injection of RAC (approximately 550 MBq; 14.86 mCi), subjects tapped their foot/fingers at their own pace in 5 blocks of 2 minutes each, with 15 seconds of rest between blocks. The UBC Research Ethics Board (following ethical standards of the Helsinki Declaration of 1975 and as revised in 1983) approved this study (certificate number: H19-03166), and written informed consent was obtained for each subject prior to all study procedures.

#### Image processing

Acquired PET data were reconstructed using PSF-HYPR4D-K-TOFOSEM,^27,28^ with attenuation, scatter, randoms, and normalization corrections, after which PET images underwent frame-to-frame realignment using SPM12.^29^ Dynamic PET images were denoised using IHYPR4D. Striatal masks were extracted by segmenting subject-specific $BP_{ND}$ images with k-means + cluster size thresholding, while cerebellar grey matter masks were extracted from FreeSurfer^30^ segmentations of structural T1 images (acquired in the same scanning session) and used to produce reference region TACs. To create group-level images, subject striatal masks were normalized to a common-space striatal mask derived from the Neurodevelopmental MRI Database (age 65–69) atlas with a 2 mm isotropic voxel size,^31^ using ANTs.^32^

#### Image analysis

To test whether our proposed method provides stronger and/or more interpretable group-level results (Aim 3 outlined in the Introduction), RSD-Hybrid-IMRTM was performed on subject-space dynamic PET images, providing β maps, while lp-ntPET was also performed, providing γ and F maps. Parametric maps were then brought to a common space using the forward transformation from the normalization step. PD subjects tapping with their left limb had their normalized images flipped. To focus on group-level differences, formal statistical testing was not performed on individual subject parameter maps; rather, raw parameter values were used to perform group-level statistical analyses.

To explore group differences in spatial DA release patterns using RSD-Hybrid-IMRTM, we performed two analyses: (i) Group-level release patterns were determined by performing one sample T-tests using β values of RSD-Hybrid-IMRTM and γ values of lp-ntPET, for each group/task combination separately, and (ii) analysis of the degree of functional segregation for HC and PD subjects was performed, comparing the overlap of subject-level foot and finger tapping parametric maps under the assumption that functional segregation would be diminished in PD thus leading to greater overlap.^33,34^ To quantify overlap the dice coefficient was computed for binarized foot and finger β and F maps across a range of sparsity thresholds. Note that T-tests made use of the full range of parameters to test for voxels with β significantly greater than zero, while the sparsity analysis only considers voxels with physically meaningful values in the context of DA release, i.e. β>0 . Sparsity subsequently ranged from 0 to 1, defined as the fraction of lowest positive β voxels removed.

To explore group differences in parameter estimation of β and γ , subject-level metrics were quantified across a range of sparsity values by computing the mean β and γ value across surviving voxels at a given sparsity threshold. For example, at sparsity s=0.8 , the mean β value was computed across the highest 20% of positive β voxels throughout the striatum, for each subject. Using sparsity for group comparison was preferable to relative thresholding of parameters (e.g. a linear threshold from $\beta /\beta_{\max}=[0\text{,}1]$ ) as the maximal β or γ value defining the range is in general sensitive to noise.

Finally the metric of “peak occupancy” ( pOcc ), inspired by recent work by Grill et al.,^23^, was evaluated using residual lp-ntPET (equation (14)) and compared to that estimated with the original lp-ntPET. pOcc provides a measure of relative binding potential changes that is comparable across subjects, defined as the maximal percentage decrease in $BP_{ND}$ caused by DA release:

(15) $$\mathrm{pOcc}=100\%*\left(\frac{BP_{ND}\left(0\right)-BP_{ND}\left(t;h=1\right)}{BP_{ND}\left(0\right)}\right)$$

where

(16) $$BP_{ND}\left(t\right)=\frac{k_{2}}{k_{2a}+\gamma h(t)}-1$$

For each subject’s foot tapping scan, pOcc was computed voxelwise using both standard and residual lp-ntPET fitting. A limited set of 5 h(t) regressors with varied $t_{P}=37–49\;\mathrm{mins}$ were used to reduce variability due to identifiability issues, similar to literature.^23^ Voxelwise distributions and mean parameter values were compared across subject groups. Note that because the residual lp-ntPET model does not contain $k_{2}$ (equation (14)), MRTM was fit to predicted baseline TACs (equation (9)) to determine $k_{2}$ and $k_{2a}$ , after which pOcc was computed. RSD β values were analyzed similarly for comparison, while γ metrics for both standard and residual lp-ntPET methods are provided in Supplemental Figure 4.

## Results

### Simulations

#### Detection performance

Results for detection sensitivity across a range of DA release scenarios are provided in Figure 2(a). RSD-Hybrid-IMRTM consistently outperforms RSD-k-means, while also proving capable of high sensitivity detection for all release scenarios. As expected, it outperforms lp-ntPET for more localized release while performing comparably for more wide-spread release.

#### Quantitative performance

Figure 2(b) compares the ability of RSD-Hybrid-IMRTM ( β ) and lp-ntPET ( γ ) to track the magnitude of the change in synaptic DA levels. Cluster-mean values in the localized left putamen cluster are displayed, while standard deviations across voxels and NRs are added in quadrature to provide error bars relevant for voxel-level quantification; results for the right putamen and left caudate clusters showed similar trends. Voxel-level histograms are also presented across all NRs.

RSD-Hybrid-IMRTM provides a metric that is more interpretable as a result of its zero y-intercept, exhibits greater linearity with $DA_{\mathrm{peak}}$ , and is also more sensitive to changes in DA concentrations, changing by a factor of 3 over the range $DA_{\mathrm{peak}}=$ 100–400 nM compared to lp-ntPET’s γ which increases by a factor of 1.5 in the same range. The non-zero y-intercept observed in the plot of γ is a result of overfitting to baseline voxel TACs, for which γ is overestimated. The longer negative tails observed in histograms of β in Figure 2(b) result from spatial blurring alongside higher contrasts of β between baseline and release voxels.

### Human scans

**Figure 3.**
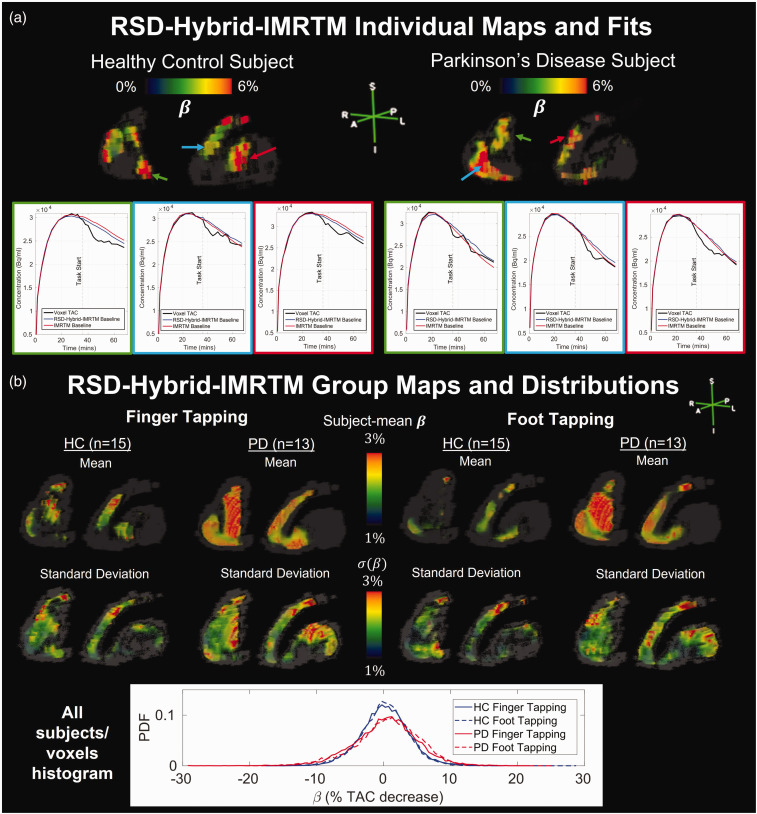
RSD-Hybrid-IMRTM outputs for individual subjects and groups. (a) Individual striatal β maps for a healthy control (HC) and Parkinson’s disease (PD) subject are displayed, along with select voxel TACs and their corresponding IMRTM and RSD-Hybrid-IMRTM fits and (b) group mean and standard deviation striatal maps of β are displayed along with voxelwise parameter distributions.

To demonstrate outputs of RSD-Hybrid-IMRTM for human subjects, Figure 3 displays β maps and voxel-level baseline TAC fits for individual subjects, along with group mean and standard deviation results.

**Figure 4.**
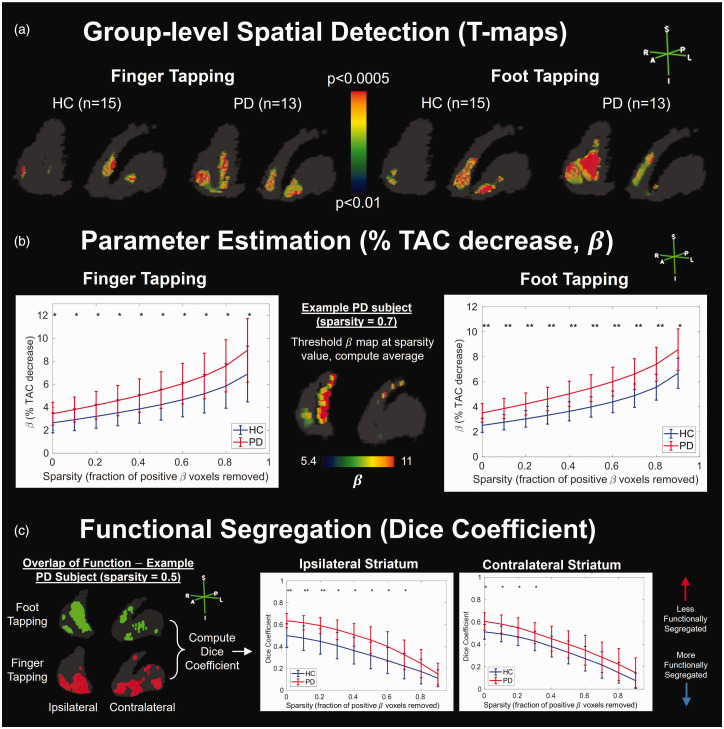
Ability of RSD-Hybrid-IMRTM to find group differences between healthy control (HC) and Parkinson’s disease (PD) subjects. (a) T-test results using subject β maps display group-level patterns of DA release. (b) Subjects’ mean β values across varied sparsity thresholds and (c) analysis of functional segregation, i.e. how distinct subject-level foot and finger tapping β maps are across varied sparsity thresholds. Sparsity is defined as the fraction of positive β voxels removed by thresholding. * p < 0.05, ** p < 0.001.

T-statistics computed separately for each group and task combination using β maps from RSD-Hybrid-IMRTM are displayed in Figure 4(a). HCs predominantly exhibit contralateral activation of localized clusters in the left putamen and caudate as may be expected of healthy brain function, with less pronounced clusters found in the ipsilateral striatum, consistent with previous work.^35^ PD subjects exhibit robust activation of large clusters in the ipsilateral striatum, which are active in both motor tasks, while contralateral activation primarily occurs in the caudate.

Computing the mean β value across voxels surviving a range of sparsity thresholds, PD subjects are found to have consistently higher parameter values for both finger and foot tapping (Figure 4(b)). While this demonstrates higher relative TAC deflection post-task in PD, note that this finding is not necessarily interpreted as PDs exhibiting higher *absolute* concentrations of DA release; see Discussion below.

Results from RSD-Hybrid-IMRTM demonstrate significantly reduced functional segregation in PD (Figure 4(c)), indicated by higher overlap between foot and finger tapping maps as measured by the dice coefficient, and is most dominant on the ipsilateral side to tapping.

Results from lp-ntPET show group differences in spatial detection that can be interpreted similarly, albeit with lower statistical power and higher spatial noise compared to RSD-Hybrid-IMRTM (Supplemental Figures 7 and 8). For group level analysis of γ values, no significant difference was found between groups.

**Figure 5.**
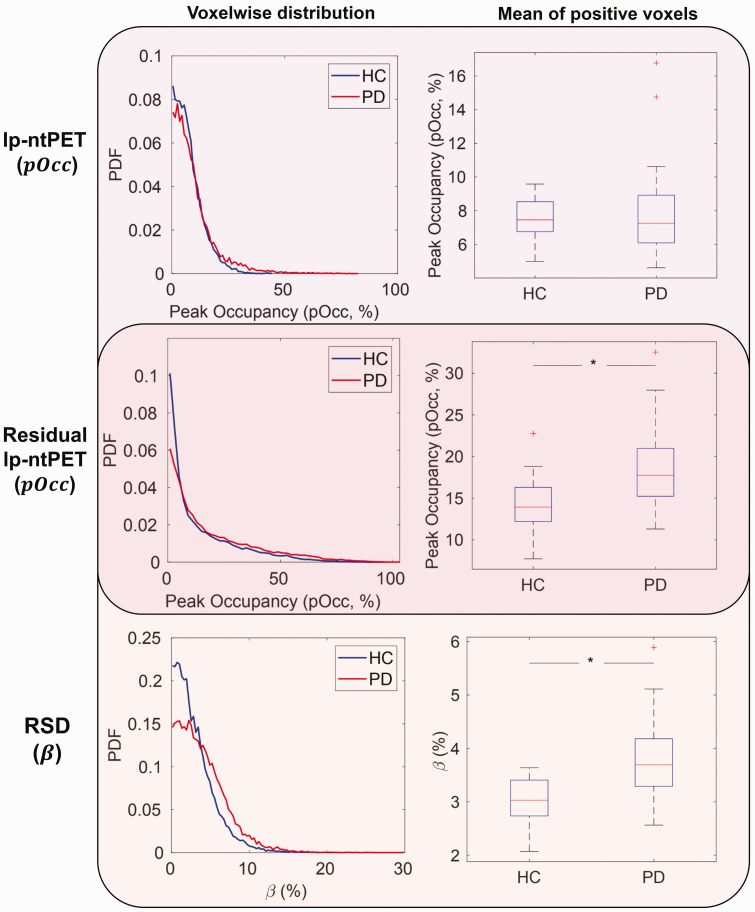
Comparison of metrics and use of residuals in humans. Peak occupancy ( pOcc ) and percentage TAC decrease ( β ) were computed for all HC and PD subjects’ foot tapping scans, across striatal voxels. The top and middle rows display lp-ntPET-based methods/metrics while the middle and bottom rows display RSD-based methods/metrics. RSD-based metrics making use of residuals find stronger group separation of parameters.

Finally, results from residual lp-ntPET are displayed in Figure 5, with comparisons to standard lp-ntPET and RSD-Hybrid-IMRTM. The top and middle rows of Figure 5 are visually grouped to compare similar lp-ntPET metrics ( pOcc ) derived separately from full TACs and residuals, while the middle and bottom rows are visually grouped to compare the use of similar residuals in computing different metrics ( pOcc and β ). Similar results are displayed for γ in Supplemental Figure 4. For our cohorts, residual analysis produces stronger group differences (Figure 5) and γ (Supplemental Figure 4).

## Discussion

### Simulations

RSD-Hybrid-IMRTM outperformed our previous approach, RSD-k-means, for all simulated scenarios of differing spatial release patterns. This improvement primarily stems from the solutions posed in Table 1, leading to more consistent and general baseline TAC predictions. lp-ntPET and RSD-Hybrid-IMRTM performed similarly for detecting global DA release as shown by the ROC curves of Figure 2(a), however localized release patterns were found more reliably with RSD-Hybrid-IMRTM, consistent with earlier results comparing lp-ntPET with RSD-k-means.^15^

Quantitative tracking of DA release was also improved by RSD-Hybrid-IMRTM. Previous work using a Bayesian framework (b-ntPET)^36^ produced similar improvements to quantitation compared with lp-ntPET, however there is an important distinction in that their work performed fitting on full-ROI TACs, while our results demonstrate that voxel-level quantitation of parameters in localized clusters can be done reliably with RSD. It is noted that release levels in this work (0–400 nM) are comparable to the range of displacement ratios from 0–10% as used in literature,^36^ which corresponds to relatively low TAC alterations, and yet these subtle changes can be differentiated with β .

While both β and γ track well with the magnitude of change in synaptic DA levels ( $DA_{\mathrm{peak}}$ )—at least in simulations—some observations on their theoretical behavior regarding release start timing ( $t_{D}$ ), receptor density ( $B_{\max}$ ), and baseline DA concentrations ( $DA_{\mathrm{baseline}}$ ) should be noted.^37^ Using noise-free simulations of varied $DA_{\mathrm{peak}}$ in the presence of variations in these parameters (Supplemental Figure 3), we found that at any given value of $DA_{\mathrm{peak}}$ , β is invariant to changes in $t_{D}$ while γ is larger for earlier task start times; both β and γ are insensitive to moderate variations in $B_{\max}$ ; and both β and γ are highly dependent on $DA_{\mathrm{baseline}}$ . This has strong implications for studying cohorts with expected differences in $DA_{\mathrm{baseline}}$ levels, such as in PD, discussed further below. Overall, observations from Figure 2(b) and Supplemental Figure 3 indicate β is a more stable metric for comparison to task- or disease-related variables that may track with relative changes in synaptic DA levels, as well as for use in group statistics.

### Human scans

#### Spatial detection

In analyzing our human cohort, RSD-Hybrid-IMRTM found significant differences between HC and PD groups for both finger and foot tapping under a variety of analyses (Figure 4). Standard lp-ntPET fitting found generally similar differences as RSD-Hybrid-IMRTM between groups (Supplemental Figures 7 and 8). However, the interpretability of results is diminished compared with RSD-Hybrid-IMRTM, with higher levels of spatial noise in T-maps and probability maps, along with lower statistical power of T-maps and dice coefficients.

While there is no ground truth validation of these observed group differences, their existence is supported by the methodological improvements to baseline TAC and DA release metric predictions made in this work. Moreover, the observed group differences are also in accordance with results from the literature regarding DA activation in HC and PD: it was hypothesized that DA release in HC should predominantly manifest in the side contralateral to tapping, with ipsilateral activation expected to be present but to a lesser extent,^35^ and this is indeed observed in the HC cohort (Figure 4(a)). PD subjects, on the other hand, primarily show activation in large clusters on their side ipsilateral to tapping, the spatial extent of which is largely similar for both tasks, possibly indicating a disease-compensatory release pattern that manifests independent of the type of motor function.^25^ This is further supported by the overlap analysis in Figure 4(c) showing reduced functional segregation in PD, primarily on the ipsilateral side, which was robust across various sparsity thresholds. An in-depth discussion and physiological interpretation of the results obtained from the human study are outside the scope of this paper; these data are part of a larger study (manuscript in preparation) and were used here only to test the performance of the proposed methods with real human data.

The issue of false positives for human studies is typically addressed on the single-subject level with a cluster size threshold (CST) which removes clusters from a binary map with fewer voxels than the CST.^7,8^ For this work we forewent use of a CST in order to preserve small clusters with the potential of group-level significance within T-maps. For the dice analysis of Figure 4(c), randomly distributed false positive clusters may systematically decrease dice coefficients, though due to their presence in all scans it is not expected to affect the interpretation of group comparisons. Moreover, at higher sparsity values it is unlikely that false positives contribute significantly as their β values are typically of lower amplitude compared with true release voxels, and thus finding significant group differences at higher sparsity levels is reassuring.

#### Utility of residual space

The determination of baseline TACs with RSD acts to isolate the DA-related signal in voxel TACs by transforming relevant information contained in TACs to a residual space. While the primary metric used in this work, β , was computed using a single residual-level regressor and GLM (equation (11)), in principle residuals can be analyzed in many ways. We implemented one such analysis by means of residual lp-ntPET (equation (14)) and found that its outputs of pOcc and γ produced significant group differences (Figure 5 and Supplemental Figure 4), whereas standard lp-ntPET fitting did not, likely stemming from the known parameter identifiability issues in lp-ntPET when simultaneously fitting to baseline and DA behavior. Future analyses could include multivariate matrix decomposition techniques applied directly to residuals, such as independent component analysis (ICA)^38^ or constrained principal component analysis (CPCA)^39^, among others. Residuals could also be input into a Bayesian estimation framework as opposed to full TACs.

#### Parameter estimation and basal DA

Considerations must be made when comparing results across different subject groups. As shown in Supplemental Figure 3, TAC-level metrics that vary with absolute DA release magnitude (e.g. β and γ ) are also affected by other physiological parameters; particularly relevant to PD cohorts, lower basal DA levels in the synapse lead to higher metric values in simulations, and this increase was observed in our PD cohort (Figure 4(b)). These group differences in β parameter magnitudes may be contributing to the generally higher T-statistics of PD subjects relative to HC (Figure 4(a)), however the spatial patterns are indeed different between groups independent of significance level. Due to the observed differences in group β values, we performed a sparsity analysis when comparing dice coefficients for Figure 4(c): sparsity ( s ) compares the fraction of the highest ( 1−s ) values of β and thus is insensitive to absolute values of β , allowing meaningful inter-group comparison. Overall, it is more appropriate to consider TAC-level metrics as measures of relative changes in synaptic DA concentrations. Ultimately, care is needed when interpreting TAC-level parameters, and comparisons to task-related metrics may need to control for disease state via additional clinical- or imaging-related metrics.^18^

## Supplemental Material

sj-pdf-1-jcb-10.1177_0271678X251362958 - Supplemental material for A generalized framework for *in vivo* detection of dopamine release using positron emission tomography Supplemental material, sj-pdf-1-jcb-10.1177_0271678X251362958 for A generalized framework for *in vivo* detection of dopamine release using positron emission tomography by Jordan U Hanania, Connor WJ Bevington, Ju-Chieh (Kevin) Cheng, Dongning Su, Alexandra Pavel, A. Jon Stoessl and Vesna Sossi in Journal of Cerebral Blood Flow & Metabolism

## Data Availability

Code to run RSD-Hybrid-IMRTM and the presented simulations are made available online (https://osf.io/PNCQY/).
