# Supplementary material for: A generalized framework for in vivo detection of dopamine release using positron emission tomography
Source: J Cereb Blood Flow Metab. 2025 Sep 19:0271678X251362958. Online ahead of print. doi: 10.1177/0271678X251362958 (PMC12449306; doi:10.1177/0271678X251362958)
Supplement: sj-pdf-1-jcb-10.1177_0271678X251362958 - Supplemental material for A generalized framework for in vivo detection of dopamine release using positron emission tomography [file sj-pdf-1-jcb-10.1177_0271678X251362958.pdf]

Supplemental Material

Jordan U Hanania[1], Connor WJ Bevington[1], Ju-Chieh (Kevin) Cheng[1,2], Dongning Su[3], Alexandra Pavel[2], A. Jon Stoessl[2,4] and Vesna Sossi[1]

1 Department of Physics and Astronomy, University of British Columbia, Canada
2 Pacific Parkinson's Research Centre, University of British Columbia, Canada
3 Center for Movement Disorders, Department of Neurology, Beijing Tiantan Hospital, Capital Medical University, Beijing, China
4 Faculty of Medicine, Division of Neurology, University of British Columbia, Canada

Corresponding author:
Jordan Hanania, UBC PET-MRI Imaging Centre, 2211 Wesbrook Mall, Vancouver, BC V6T2B5 Canada
Email: jhanania@phas.ubc.ca

Table of Contents

List of Symbols 3
Quantitative behavior 5
Variation of γ with choice of h(t) 5
Effect of non-local means kernel size (h^2) 5
Theoretical metric behavior vs other physiological parameters 6
Residual lp-ntPET 7
Quantitative behavior (cont.) 9
Varied DA release magnitude parameter histograms (20 noisy realizations) 9
Human results from lp-ntPET 10
Simulation details 12
Iterative MRTM (IMRTM) 17
Convergence of IMRTM 17
Demonstration of IMRTM for Simulated Data 18
Comparison to different baseline methods in RSD 20
References 22

List of Symbols

Below is a list of symbols used in the main text, in order of appearance in the text. References to their definitions are provided when applicable, denoting the main text equation where they were defined.

Symbol	Description	Definition
t	Time post tracer injection; minutes	Eq. 1
$C_T(t)$	Target voxel/region tissue concentration at time t; Bq/ml	
$C_R(t)$	Reference region (cerebellum) tissue concentration at time t; Bq/ml	
R_1	Relative tracer delivery rate, $K_1/K_1{}'$; unitless	
k_2	Tracer efflux rate constant; s-1	
k_{2a}	Apparent tracer efflux rate constant; s-1	
γ	Magnitude of neurotransmitter release modulation to $k_{2a}(t)$; s-1	
$h(t)$	Temporal shape of the neurotransmitter release modulation to $k_{2a}(t)$; unitless, $\max(h) = 1$	Eq. 2
t_D	Task start time; minutes	
t_P	Time of maximal tracer efflux rate; minutes	
α	Sharpness of $h(t)$ curve; unitless	
$\theta(t - t_D)$	Heaviside step function; unitless	
$C_{IMRTM}^{(n)}$	n^{th} baseline TAC prediction from IMRTM; Bq/ml	Eq. 4
$\tilde{R}_1, \tilde{k}_2, \tilde{k}_{2a}$	Z-scored (mean-subtracted, standard deviation scaled) MRTM parameters; unitless	-
$w_{i,j}$	Non-local means (NLM) weight between voxels i and j, indicating similarity of pre-task-derived MRTM kinetics; unitless	Eq. 5
h^2	Hyperparameter to define the NLM neighborhood; unitless	
$\vec{d}_n$	Position of voxel n in z-scored MRTM parameter space; unitless	
F_i	F-statistic for voxel i, computed by comparing lp-ntPET and MRTM fits to the measured voxel TAC; unitless	-
$\tilde{C}(t)$	Baseline TAC regressor set, containing measured “baseline” TACs and IMRTM-derived baseline TACs; Bq/ml	Eq. 6
$C_{NLM,j}(t)$	NLM-derived baseline TAC for voxel j; Bq/ml	Eq. 7
$\hat{C}_j(t)$	RSD-Hybrid-IMRTM-derived baseline TAC for voxel j; Bq/ml	Eq. 9
$R_{pct,j}(t)$	Percentage residuals calculated as the percent difference between the RSD-derived baseline TAC and measured TAC for voxel j; %	Eq. 10,11
$P(t)$	Predicted shape of percentage residuals; unitless, $\max(P) = 1$	Eq. 11,12
$R_{abs}(t)$	Absolute residuals calculated as the difference between the RSD-derived baseline TAC and measured TAC; Bq/ml	Eq. 13

BP_{ND}	Non-displaceable binding potential; unitless	Eq. 15,16
$pOcc$	Maximal percentage decrease in BP_{ND} caused by neurotransmitter release; unitless	

Quantitative behavior

Variation of γ with choice of $h(t)$

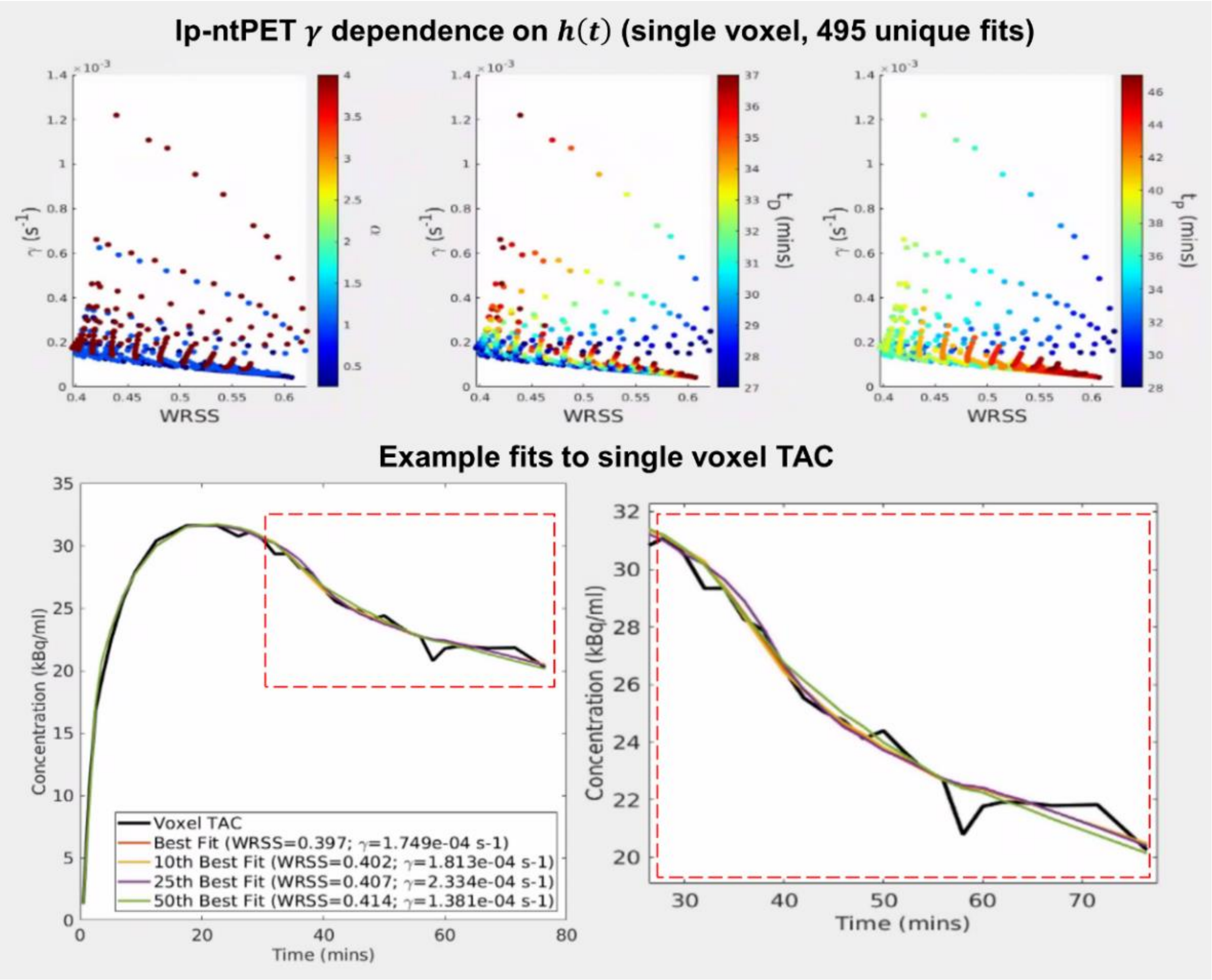

Supplemental Figure 1. Demonstration of variability in γ dependent on the choice of $h(t)$. 495 unique $h(t)$ basis functions were fit, each providing a TAC fit with its own weighted residual sum of squares (WRSS) shown in the top row vs γ. The bottom row displays similar TAC fits and their respective WRSS and γ values.

Effect of non-local means kernel size (h^2)

The effect of NLM kernel size h^2 (main text Equation 5) was tested for the mid-sized release simulations using the RSD-Hybrid-removal configuration. h^2 was set to 0.25, 0.5, 1, and 2 (units of standard deviation), with ROC curves produced across 50 noisy realizations. Small kernel sizes $h^2 = 0.25$ and $h^2 = 0.5$ performed nearly identically, while detection performance degraded with larger kernel size. This is due to the progressive inclusion of voxels further away in kinetic space, which reduces the concordance of pre-task behaviors for the predicted baseline TAC and measured voxel TAC. Kernel size of $h^2 = 0.5$ was used throughout this work.

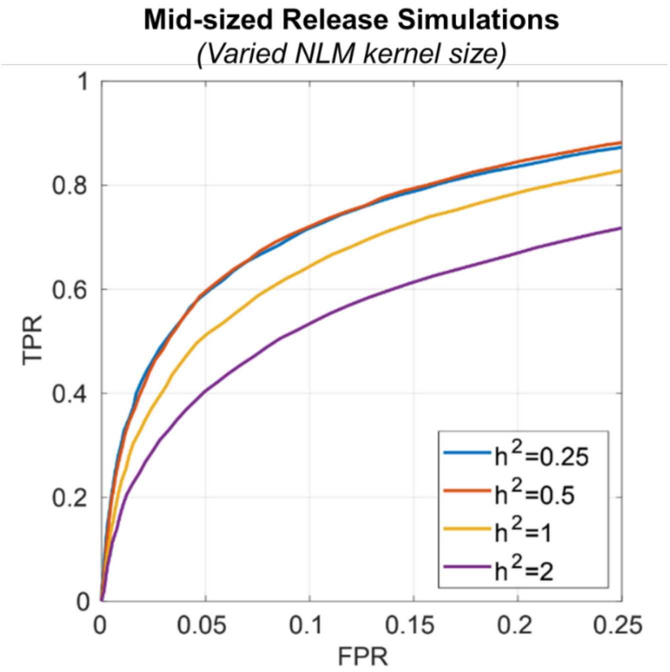

Supplemental Figure 2. Detection performance for low-amplitude DA release in mid-sized clusters (50 noisy realizations), comparing different kernel sizes h^2 used in RSD-Hybrid-IMRTM. True positive rate (TPR) is calculated across all ground truth clusters, while false positive rate (FPR) is calculated across the release-free right caudate region. Note, the abscissa is limited to the range 0-25% FPR to highlight methodological differences.

Theoretical metric behavior vs other physiological parameters

To examine the theoretical behavior of RSD and lp-ntPET metrics with DA release magnitude and other physiological parameters, 10,000 noise free simulations were produced with randomized parameters of DA release concentration (DA_{peak}), task start time (t_D), receptor density (B_{max}), and baseline DA concentration ($DA_{baseline}$) (Supplemental Figure 3).

At any given value of DA_{peak}, β is invariant to changes in t_D while γ is larger for earlier task start times; both β and γ are insensitive to modest variations in B_{max}; and both β and γ are highly dependent on $DA_{baseline}$. This is expected as both methods evaluate relative changes in synaptic dopamine levels – for equal release the TAC change will be greater in a situation of lower baseline dopamine levels. This must be kept in mind when comparing results in populations that are expected to have differences in baseline DA levels, such as PD and controls.

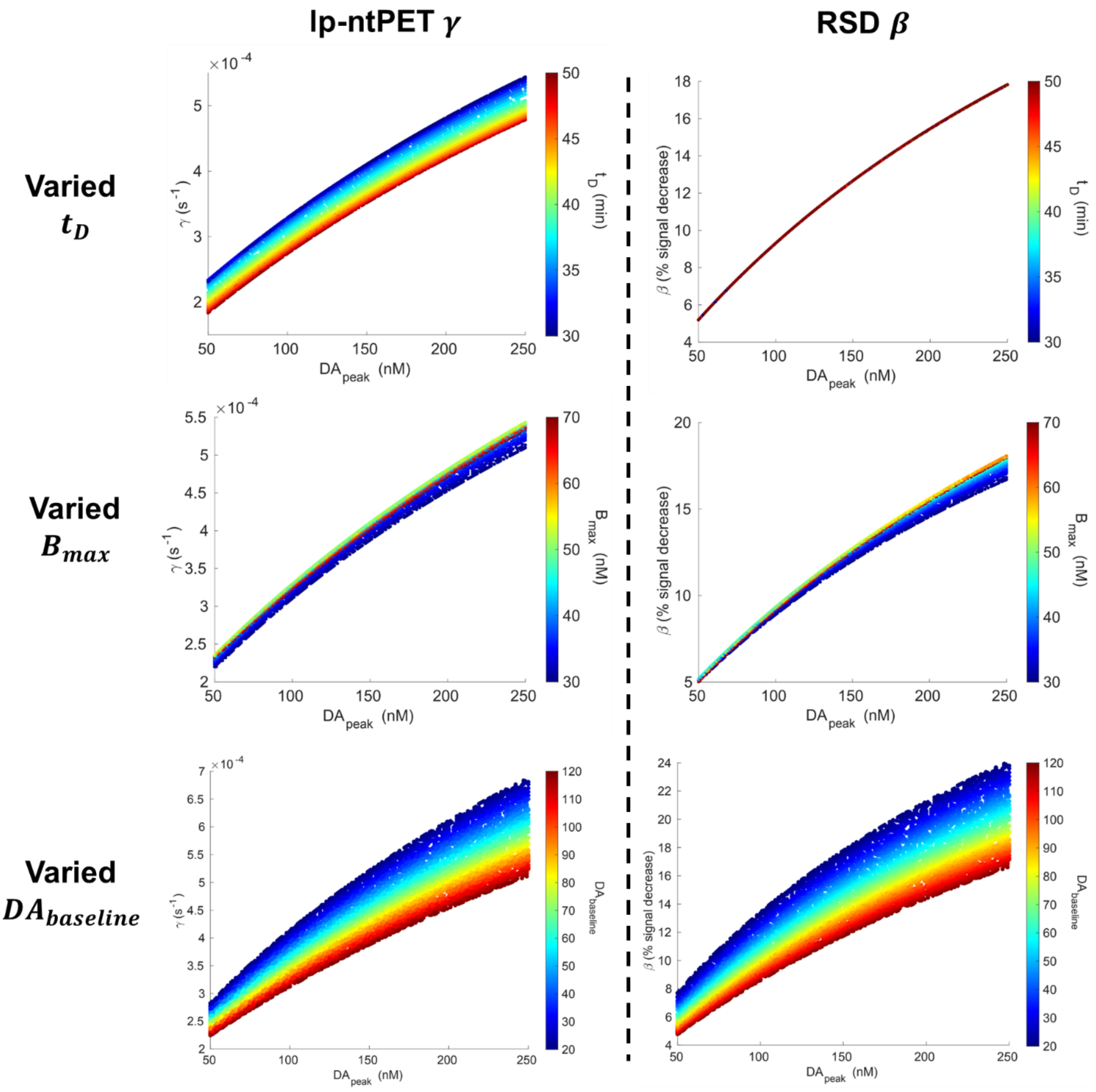

Supplemental Figure 3. Testing robustness of parameters γ and β to simultaneous variations in important physiological parameters while tracking absolute synaptic DA concentration changes (noise free).

Residual lp-ntPET

Figure 4 displays results for residual lp-ntPET applied to the human cohorts described in the main text in regard to the parameter γ. Compared with standard lp-ntPET, residual lp-ntPET's γ estimates show significant group differences. See the main text for more detail.

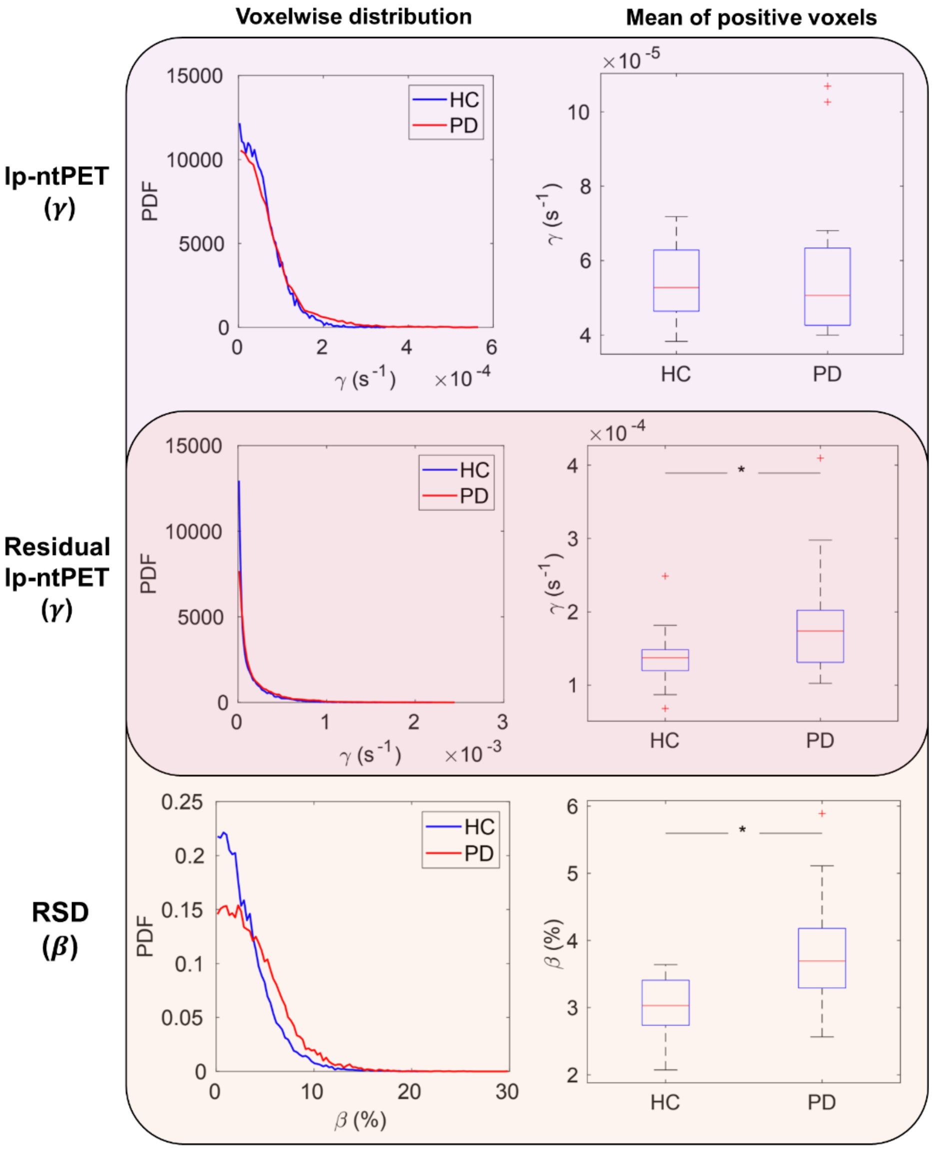

Supplemental Figure 4. Comparison of metrics and use of residuals. γ and β were computed for all HC and PD subjects, across striatal voxels. The top two rows display lp-ntPET-based methods/metrics while the bottom two rows display RSD-based methods/metrics. RSD-based metrics making use of residuals find stronger group separation of parameters.

Figure 5 displays an example fit of the residual lp-ntPET model (main text Eq. 13) to a voxel's residuals.

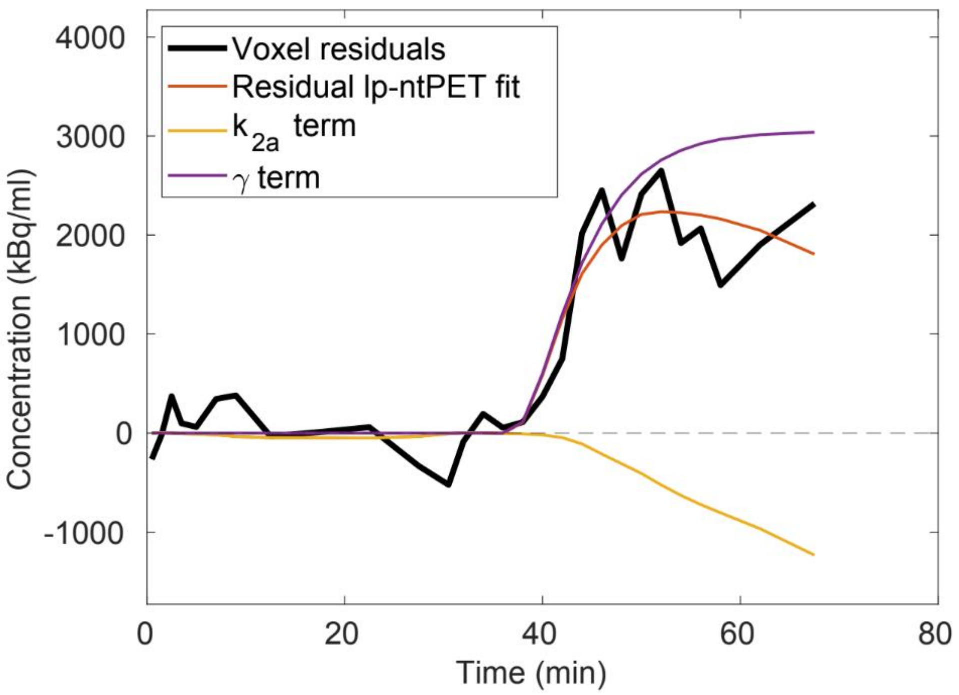

Supplemental Figure 5. Example residual lp-ntPET fit for a single voxel of a human subject performing foot tapping. The task began at 37 minutes post-injection and 5 $h(t)$ regressors were used for model selection with fixed $t_D = 37$ min, $\alpha = 1$, and t_P varied.

Quantitative behavior (cont.)

Varied DA release magnitude parameter histograms (20 noisy realizations)

The main text displays the quantitative metrics' ability to track with DA release for both lp-ntPET and RSD-Hybrid-lMRTM, however results displayed cluster-mean values and standard deviations for the left putamen cluster alone. Here we display histograms for each simulated dataset for both γ and β, for all clusters and baseline voxels across 20 noisy realizations (Supplemental Figure 6). Both methods' metrics show gradual separation with increasing DA release magnitudes, however lp-ntPET's γ shows a generic offset from 0 for baseline voxels as shown by the median line of baseline voxels.

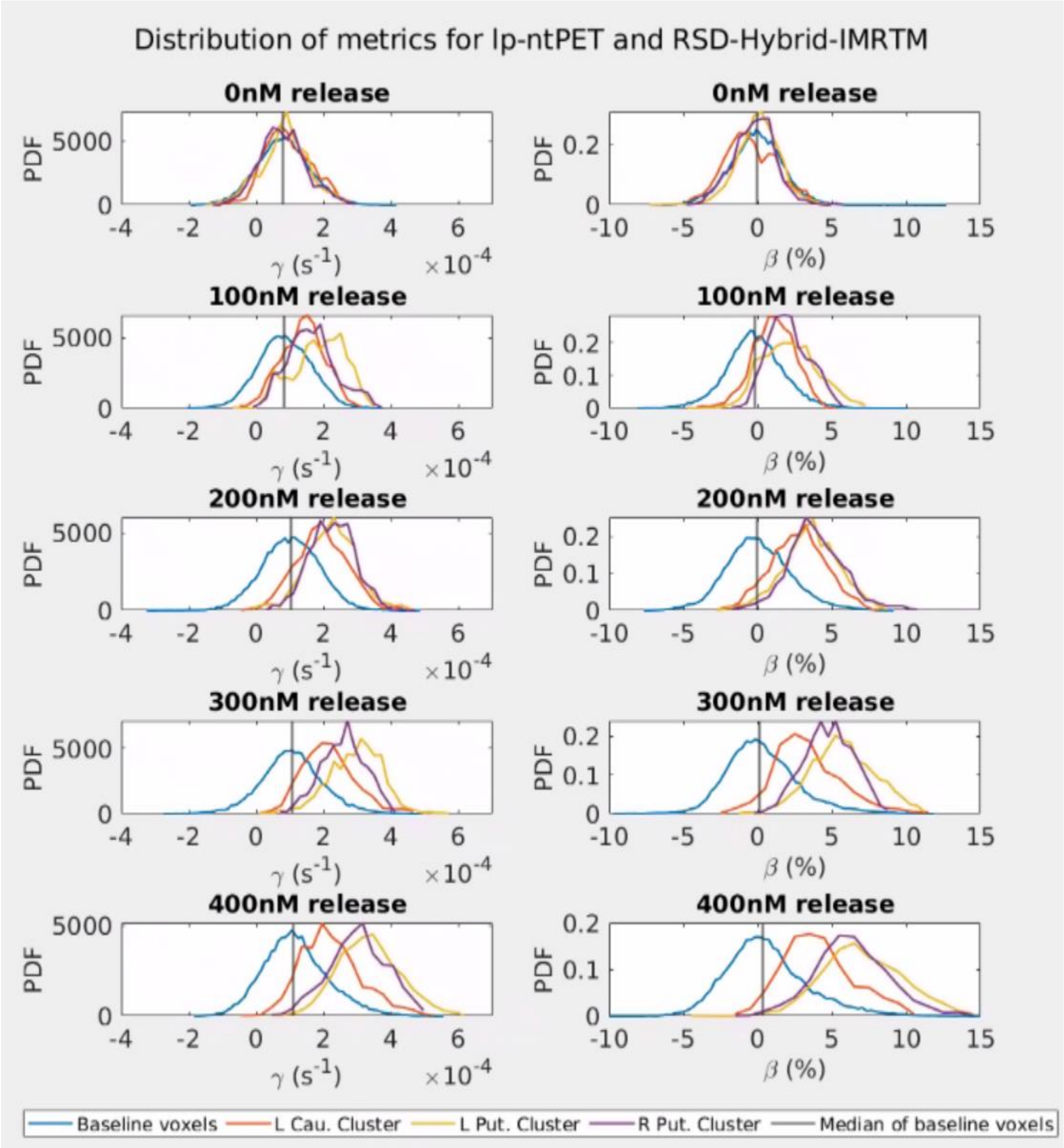

Supplemental Figure 6. Probability density functions (PDF) for metric values from simulations of varied DA release for lp-ntPET (γ, left column) and RSD-Hybrid-IMRTM (β, right column).

Human results from lp-ntPET

Results from lp-ntPET analysis for the human cohorts described in the main text are provided here (Supplemental Figures 7 and 8). Analyses mimic those of RSD-Hybrid-IMRTM in Figure 4 of the main text, with the addition of "probabilistic maps" computed from thresholded F-statistic maps (similar to previous works, see [1,2]), interpretable as the percentage of subjects that had a given voxel in their thresholded F-map.

Supplemental Figure 7 displays one-sample T-test maps computed using unthresholded γ (Supplemental Fig 5a). Due to the variance of γ with choice of $h(t)$ (Supplemental Figure 1) γ values used in Supplemental Figures 7a and 8a are derived from the use of a single $h(t)$, with $\alpha = 1$, $t_D = 36$ mins, and $t_P = 42$ mins. Conversely, detection using the F-statistic (Supplemental Figures 7b and 8b) make use of lp-ntPET fitting with 495 different choices of $h(t)$.

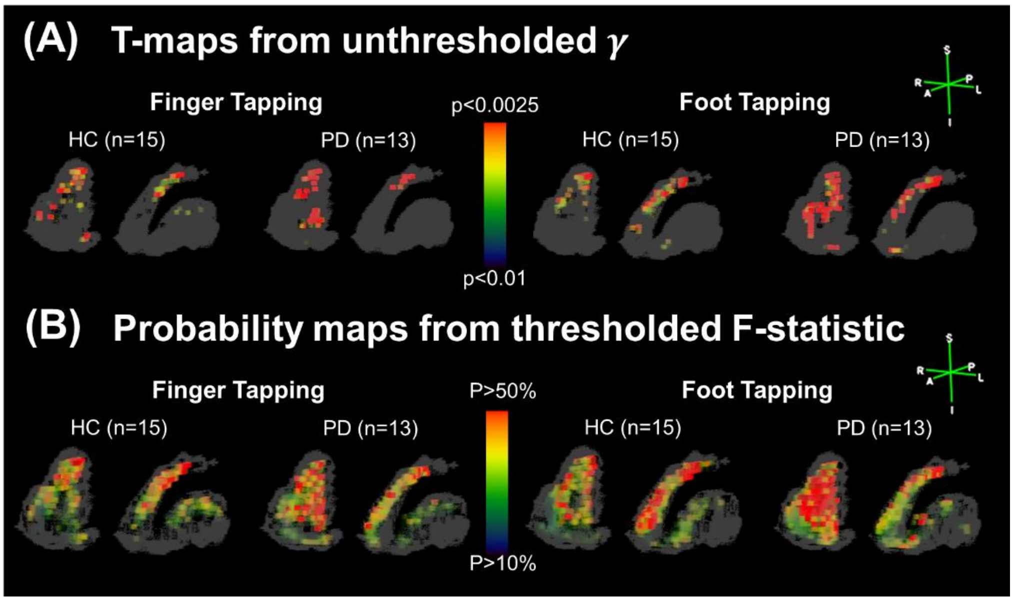

Supplemental Figure 7. Group-level patterns of DA release found with lp-ntPET for healthy control (HC) and Parkinson's disease (PD) subjects. (A) T-test results using subject γ maps. (B) Probability maps computed using subject F-statistic maps thresholded at $p(F) < 0.05$.

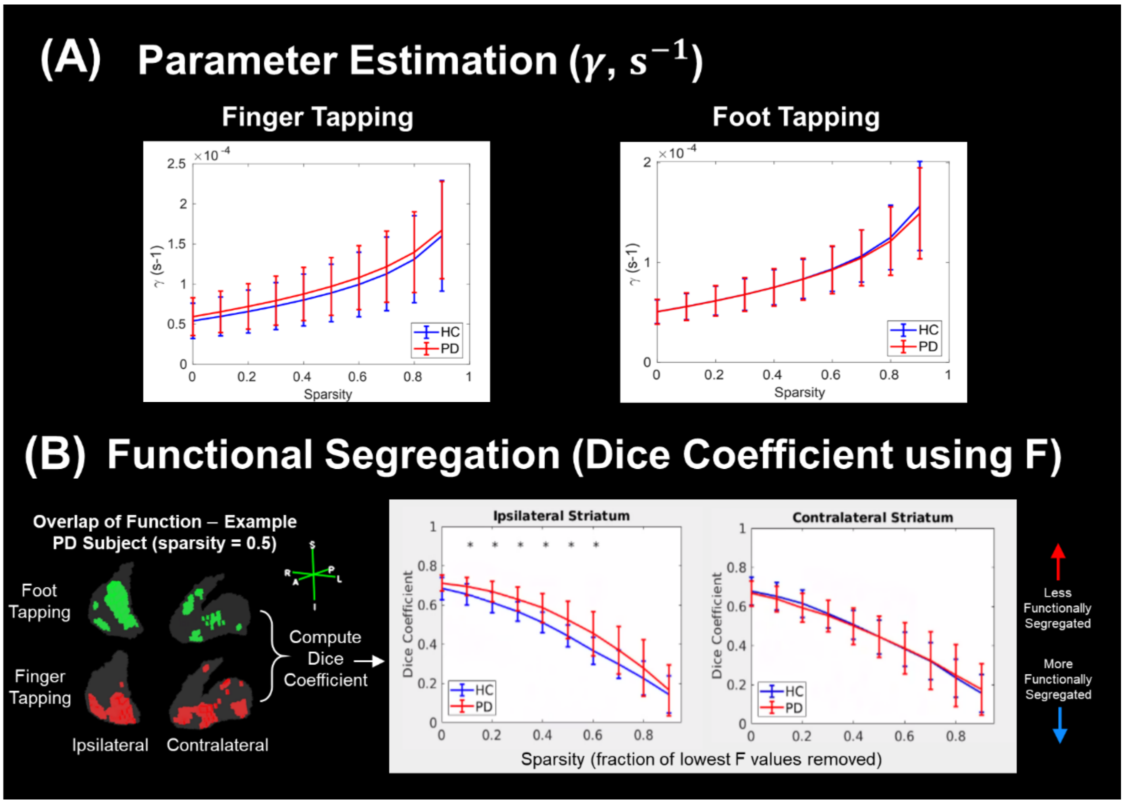

Supplemental Figure 8. Additional group-level results from lp-ntPET for healthy control (HC) and Parkinson's disease (PD) subjects. (A) Subjects' mean γ values across varied sparsity thresholds. (B) Analysis of functional segregation, i.e. how distinct subject-level foot and finger tapping F-statistic maps are across varied sparsity thresholds; * indicates p<0.05.

Simulation details

We simulated 79-minute single-task RAC scans on the GE SIGNA PET/MR system (GE Healthcare, Chicago, IL, USA). A T1 MRI from a healthy human subject scanned on the SIGNA was segmented using FreeSurfer[3] to generate an anatomical reference for simulating regions with pre-determined RAC kinetics; the segmented image was then resampled to the PET voxel dimensions (1.39x1.39x2.78 mm) using nearest-neighbor interpolation. The striatum was defined using bilateral ROIs from the putamen, caudate, and nucleus accumbens.

Noiseless TACs were simulated following the ntPET model,[4] using the same approach as Wang *et al*. to simulate DA release.[5] Parameters for the simulations were derived from a single 60 minute RAC scan of a healthy control subject under baseline conditions, following the reference region approach of the original ntPET work.[4] An eroded striatal ROI was used to derive a single mean TAC for fitting purposes (Supplemental Figure 9, blue TAC). The parameter R_1 was fixed to 1.0055 by first fitting MRTM, while DA kinetic parameters k_{on}^{DA} and k_{off}^{DA} were assumed at 0.25 ml/(pmol*min) and 25 min-1, respectively.[4,6]

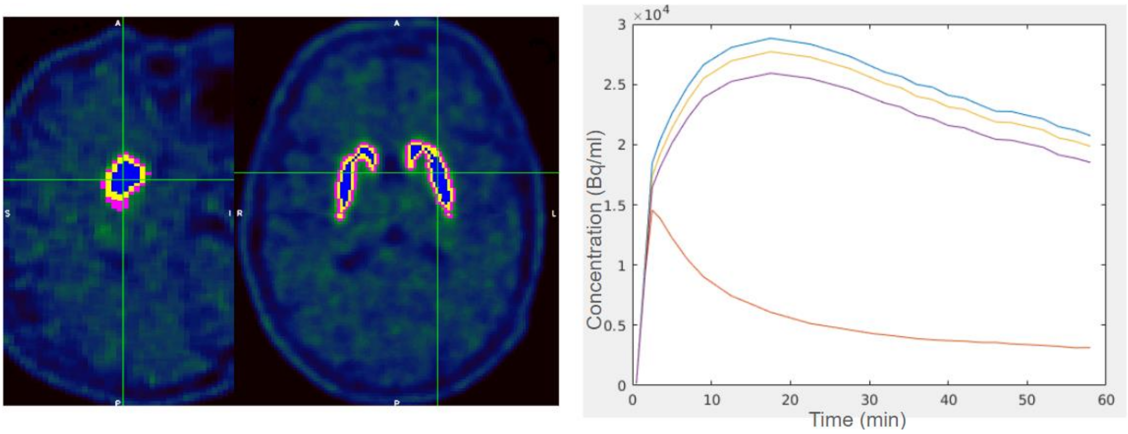

Supplemental Figure 9. Single RAC frame for healthy control subject with striatal ROIs overlaid. ROI color corresponds to TAC color, and the orange TAC is the mean cerebellum TAC. The blue eroded ROI TAC was used for ntPET fitting.

Parameter	ntPET[4] Fit to Baseline TAC (Blue TAC of Supp. Fig. 9)	Literature (Pappata[7])
K_1	0.0872 ml/min/ml	0.0918 ml/min/g
k_2	0.5765 min-1	0.4484 min-1
k_{off}	0.1022 min-1	0.1363 min-1

k_{on}	0.02719 min-1/(pmol/ml)	0.0282 min-1/(pmol/ml)
$B_{\max}$	33 pmol/ml	44 pmol/ml
Basal DA	96.4 pmol/ml	100 pmol/ml

Supplemental Table 1. Simulation parameters for use in the ntPET model.

Motivated by observed binding heterogeneities in subjects with PD (Supplemental Figure 11) we simulated a gradient in the binding potential (BP), accomplished by modulating $B_{\max}$, where

$$B_{\max} = K_D\, BP \qquad (1)$$

and K_D is the equilibrium dissociation constant.[7] $B_{\max}$ alone enters the ntPET model and so K_D and BP do not need to be assumed. The gradient was designed to approximate the direction and magnitude of observed concentration gradients in human data. The simulated B_{max} ranged from 70 pmol/ml in the superior and posterior regions of the striatum, to 30 pmol/ml in the anterior and inferior regions, with a slope of -30° in the anterior direction.

The TACs were framed 4x1, 3x2, 4x5 minutes for estimation of the baseline portion of the scan, then 20x2 minutes for higher temporal sampling during DA release, and finally 1x4, and 2x5 minutes at the end of the scan for better counting statistics. Dynamic images were created by placing DA release TACs into specified clusters within the striatum (see below) and baseline TACs into all other striatal voxels. The dynamic images were then spatially filtered with a 1.8 x 1.8 x 3.6 mm FWHM Gaussian to simulate the SIGNA point spread function (PSF).[8] Noisy images (NRs) were produced using scaled Gaussian noise, before post-processing with IHYPR4D (7.2 mm, two frames spatiotemporal kernel, one iteration);[9] the scaling coefficient was set so that the noise level after applying IHYPR4D matched human RAC scan noise levels reconstructed with PSF-HYPR4D-K-TOFOSEM (5.6 mm, ten frames spatiotemporal kernel, 10 iterations)[10] and post-processed with IHYPR4D. Noise level σ is added to concentrations at each frame i, $C_T(t_i)$, given by:[11]

$$\sigma_i = \mu \times e^{\lambda t_i} \times \sqrt{C_T(t_i)e^{-\lambda t_i}/\Delta t_i} \qquad (2)$$

where the scaling factor μ determines noise level, λ is the Carbon-11 decay constant, and Δt_i is frame i's duration. Choosing $\mu = 1.35$ in combination with denoising by IHYPR4D matched with human scan noise levels.

Clusters of DA release were defined as follows: the localized and mid-sized clusters were defined using varying sized bounding boxes conjoined with non-edge striatal voxels, while full-ROI clusters were non-edge left caudate, left putamen, and right putamen voxels (main text Figure 2a).

Simulating the DA release timecourse

The simulations aim to provide a realistic spatiotemporal release pattern in response to a motor/cognition control task. The following task protocol is used to guide the simulation of DA release timecourse and is based on the real task presented to the human subject of this study. After task start, subjects tapped their fingers for 10 minutes, in blocks of 2 minutes with 15 seconds of rest between blocks. The task involved serial tapping of the index finger, middle finger, ring finger, and little finger, then reversed ('1-2-3-4, 4-3-2-1'…), at the subject's own pace.

The spatiotemporal progression of extracellular DA concentration has been modeled as follows:[1]

$$D_e(r,t) = \sum_{i,j(t\geq t_{i,j})} \frac{\ell_s V_s C_s}{\alpha(4\pi D(t-t_{i,j}))^{3/2}} \exp\left[-\frac{(r-r_i)^2}{4D(t-t_{i,j})} - k(t-t_{i,j})\right] \qquad (3)$$

where i indexes over synapses, j indexes over release times, V_s is the Volume of the synapse, C_s the DA concentration within the synapse, D the apparent diffusion coefficient, α the fraction of extracellular volume, ℓ_s the fraction of synaptic leakage or spillover, and k is the effective removal rate constant. The average extracellular DA at any point in time within a PET voxel is then the integration of D_e over the volume of the voxel, divided by the volume of the voxel. For simplicity, we assume a uniform distribution of synapses within the voxel that all fire at the same time, and model a PET voxel as a spherical volume with radius a. Letting

$$A_j(t) = \frac{\ell_s V_s C_s}{\alpha(4\pi D(t-t_{i,j}))^{3/2}}, \qquad (4)$$

we have:

$$\langle D_e\rangle_a = \frac{1}{\frac{4}{3}\pi a^3}\int_0^a D_e(r,t) 4\pi r'^2 dr' \qquad (5)$$

$$= \frac{1}{\frac{4}{3}\pi a^3}\sum_j A_j(t)\int_0^a \exp\left[-\frac{r'^2}{4D(t-t_{i,j})}\right] dr' \qquad (6)$$

$$= \frac{3D}{a^3}\sum_j A_j(t)\cdot(t-t_j)\left[\sqrt{4\pi D(t-t_j)}\,\mathrm{erf}\left(\frac{a}{\sqrt{4\pi D(t-t_j)}}\right) - 2a\exp\left(-\frac{a^2}{4D(t-t_j)}\right)\right] \qquad (7)$$

Typical striatal values of *D* are on the order of 10^{-6} cm^2/s, and for most PET scanners a is on the order of 0.1 cm.[12] Therefore for t on the order of t_j, $\mathrm{erf}\left(\frac{a}{\sqrt{4\pi D(t-t_j)}}\right) \to 1$ and $\exp\left(-\frac{a^2}{4D(t-t_j)}\right) \to 0$. As a result:

$$\langle D_e\rangle_a \approx \frac{3}{a^3}\sum_j \frac{\ell_s V_s C_s}{4\pi\alpha}\exp[-k(t-t_j)] \qquad (8)$$

Returning to our series of task events described above, we thus model the time-varying DA concentration fed to the ntPET model as

$$F_{DA}(t) = b + \sum_j g_j \theta(t - t_j) \exp\left(-\frac{t - t_j}{\tau_j}\right) \quad (9)$$

where b is the basal DA concentration of 100 pmol/mL, j indexes over each task event response, g_j scales the magnitude of the jth response, and τj controls the decay rate of the jth response. Cognition- and motor-based response is assumed to occur every 20 seconds during each 10-minute task block.

g and τ are kept constant for each response within a class of response; empirically we set $g_{motor} = g_{cognitive} = 34$ pmol/ml and $\tau_{motor} = \tau_{cognitive} = 60$ s to yield ~100% increase from basal for the motor and cognitive clusters. See Supplemental Figure 10 for the temporal release profiles for a sample realization.

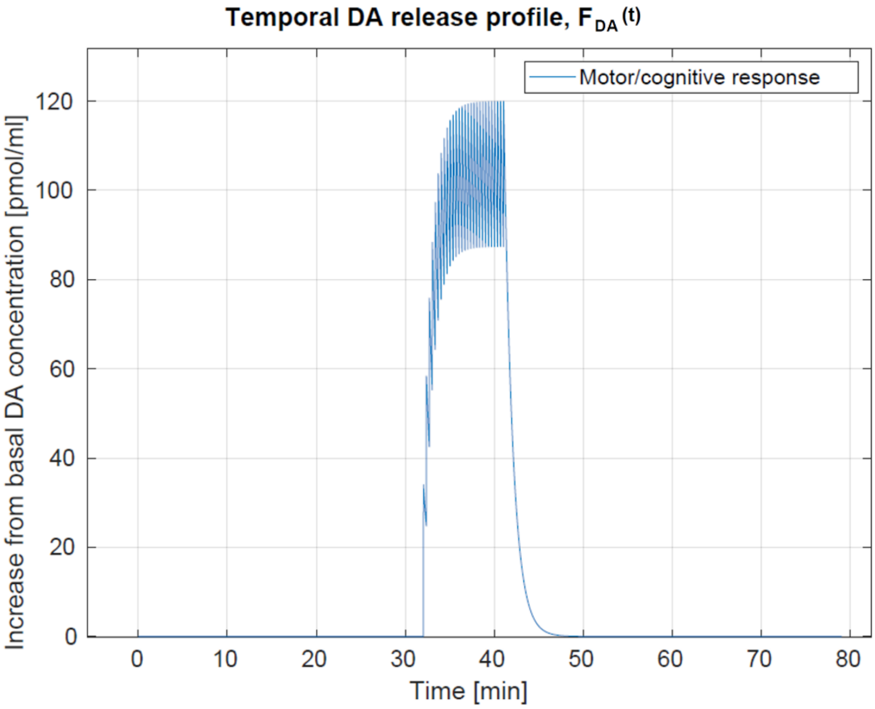

Supplemental Figure 10. Temporal DA release profile ($F_{DA}(t)$) for simulated motor task performance. The numerical curve is fed to the ntPET model to generate noiseless DA release TACs. Due to the PET temporal resolution on the order of minutes, the sawtooth pattern present in $F_{DA}(t)$ does not appear in the noiseless TACs.

Heterogeneous binding

Observed gradients in the striatum in Parkinson's disease

The spatially heterogeneous binding observed in Supplemental Figure 11 motivated the need for heterogeneity modeling of TACs within RSD. Healthy subjects typically show relatively homogeneous tracer binding within the striatum, however Parkinson's subjects exhibit spatial variations that need to be accounted for.

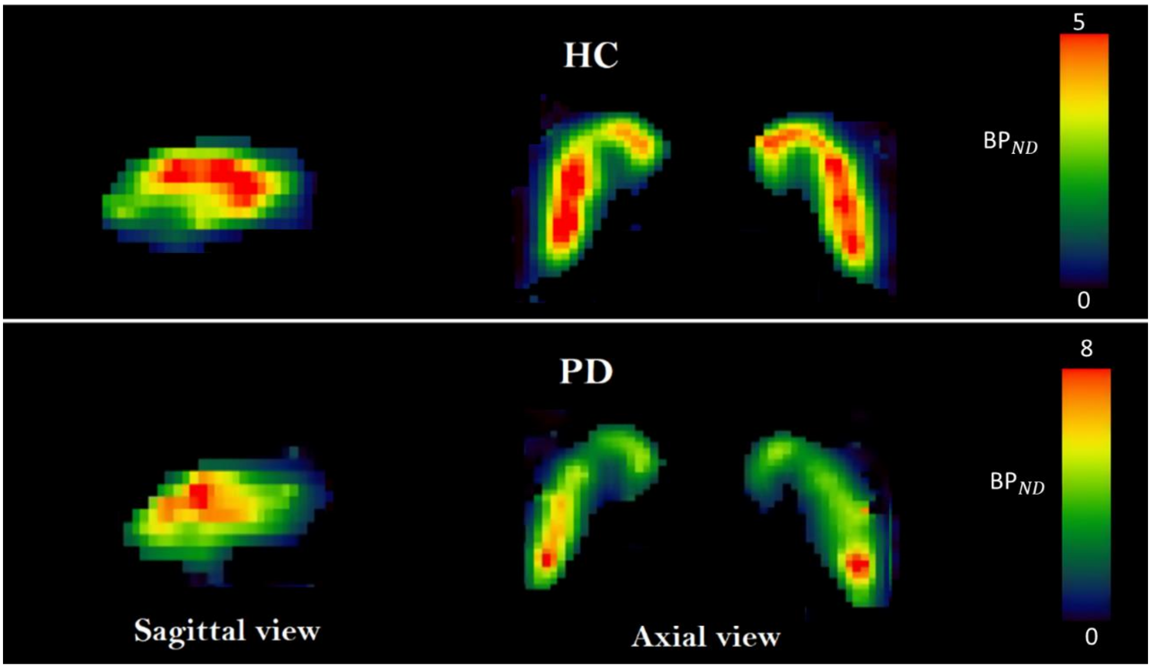

Supplemental Figure 11. Striatal BP_{ND} values computed using MRTM for a healthy control (HC) and Parkinson's disease (PD) subject. These images were reconstructed with PSF-HYPR4D-K-TOFOSEM.

Simulations were performed as stated above: "The gradient was designed to approximate the direction and magnitude of observed concentration gradients in human data. The simulated B_{max} ranged from 70 pmol/ml in the superior and posterior regions of the striatum, to 30 pmol/ml in the anterior and inferior regions, with a slope of -30° in the anterior direction." The simulated B_{max} gradient images were used in ntPET modeling to generate 4D PET concentration images. An example noise-free concentration volume is displayed in Figure 12 below.

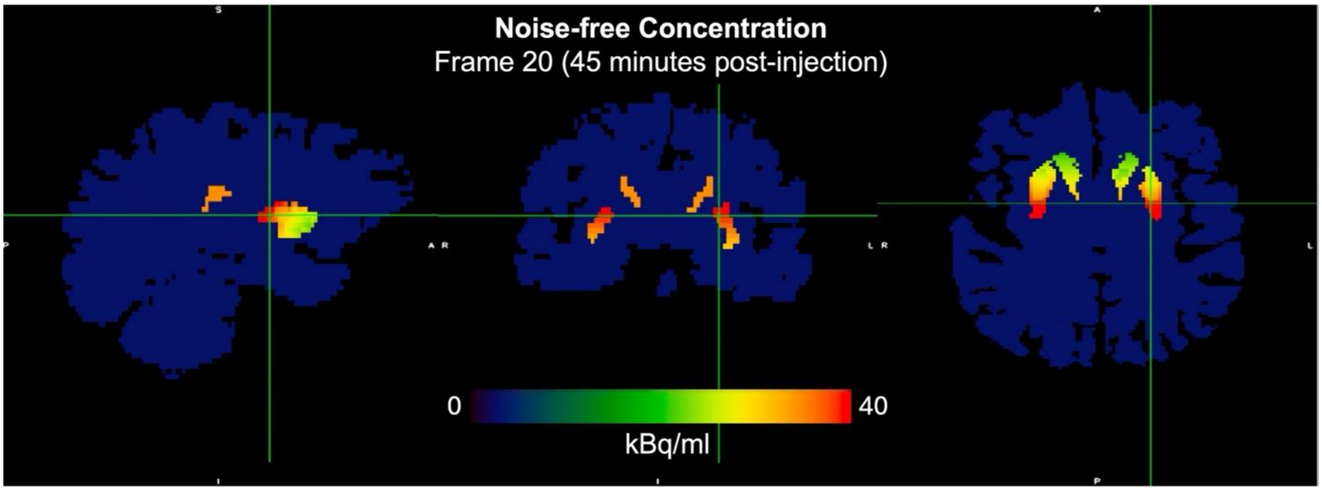

Supplemental Figure 12. Simulated noise-free concentration image (frame 20, 45-minutes post-injection) used to simulate heterogeneous RAC binding. Method performance in the main text was tested on noisy versions of these heterogeneous binding simulations.

Iterative MRTM (IMRTM)

Convergence of IMRTM

From the main text, the IMRTM equation for the nth-iteration baseline TAC prediction was

$$C_{IMRTM}^{(n)}(t) = R_{1,pre}C_R(t) + k_{2,pre}\int_0^t C_R(u)du - k_{2a,pre}\int_0^t C_{IMRTM}^{(n-1)}(u)du \qquad (10)$$

To see why IMRTM converges towards a baseline TAC, consider a measured voxel TAC to be composed of a baseline term $C_B(t)$ (which is well described by MRTM) and a release term $R(t)$

$$C_{IMRTM}^{(0)}(t) = C_T(t) = C_B(t) - R(t) \qquad (11)$$

Substituting Eq. 11 into Eq. 10, we get

$$\begin{aligned} C_{IMRTM}^{(1)}(t) = R_{1,pre}C_R(t) + k_{2,pre}\int_0^t C_R(u)du - k_{2a,pre}\int_0^t C_B(u)du \\ + k_{2a,pre}\int_0^t R(u)du \end{aligned} \qquad (12)$$

The first 3 terms can be simplified to $C_B(t)$ as a result of MRTM being an adequate model for the baseline TAC:

$$C_{IMRTM}^{(1)}(t) = C_B(t) + k_{2a,pre}\int_0^t R(u)du \qquad (13)$$

This first estimate approximates the baseline TAC $C_B(t)$ with positive bias post-task proportional to the integral of the release term. Substituting Eq. 13 into the right-hand side of Eq. 10 and repeating the simplification of MRTM terms gives the estimate of iteration 2

$$\begin{aligned} C_B^{(2)}(t) &= R_{1,pre}C_R(t) + k_{2,pre}\int_0^t C_R(u)du - k_{2a,pre}\int_0^t C_{IMRTM}^{(1)}(u)du \\ &= R_{1,pre}C_R(t) + k_{2,pre}\int_0^t C_R(u)du - k_{2a,pre}\int_0^t C_B(u)du \\ &\quad - \left(k_{2a,pre}\right)^2 \iint_0^t R(u)du^2 \\ &= C_B(t) - \left(k_{2a,pre}\right)^2 \iint_0^t R(u)du^2 \end{aligned} \qquad (14)$$

Similarly, iteration 3 is given by inserting the final line of Eq. 14 into Eq. 10:

$$\begin{aligned} C_B^{(3)}(t) &= R_{1,pre}C_R(t) + k_{2,pre}\int_0^t C_R(u)du - k_{2a,pre}\int_0^t C_{IMRTM}^{(2)}(u)du \\ &= R_{1,pre}C_R(t) + k_{2,pre}\int_0^t C_R(u)du - k_{2a,pre}\int_0^t C_B(u)du \\ &\quad + \left(k_{2a,pre}\right)^3 \iiint_0^t R(u)du^3 \end{aligned} \qquad (15)$$

$$= C_B(t) + \left(k_{2a,pre}\right)^3 \iiint_0^t R(u)du^3$$

Iterating this procedure results in

$$C_{IMRTM}^{(n)}(t) = C_B(t) - \left(-k_{2a,pre}\right)^n \int_n R\,(u)du^n \qquad (16)$$

with $\int_n$ denoting n repeated integrals from 0 to t. Thus $C_{IMRTM}^{(n)}(t) \approx C_B(t)$ if the condition $\lim_{n\to\infty}\left(k_{2a,pre}\right)^n \int_n R\,(u)du^n = 0$ is satisfied.

To evaluate this term's convergence, the MRTM equation can be rearranged for k_{2a},

$$k_{2a} = \frac{R_1 C_R(t) + k_2 \int_0^t C_R(u)du - C_T(t)}{\int_0^t C_T(u)du} \qquad (17)$$

In the large-n limit of $\left(k_{2a,pre}\right)^n$, it is noted that the dominant term $\left(\int_0^t C_T(u)du\right)^n$ appears in the denominator, thus $\left(k_{2a,pre}\right)^n \sim \left(\int_0^t C_T(u)du\right)^{-n}$ (ignoring units).

The nth repeated integral of a function in the interval $[0,t]$ may be rewritten using the Cauchy repeated integral formula, as

$$\int_n R\,(u)du^n = \frac{1}{(n-1)!}\int_0^t (t-u)^{n-1} R(u)du \qquad (18)$$

Substituting Eqs. 17 and 18 into the convergence condition,

$$\lim_{n\to\infty} \frac{\int_0^t (t-u)^{n-1} R(u)du}{(n-1)!\left(\int_0^t C_T(u)du\right)^n} = 0 \qquad (19)$$

which will be satisfied for all practical cases due to the $(n-1)!$ in the denominator. Mathematically, divergence may occur if $C_T(t) \to 0$; such a scenario is non-physical however, as in the limit $k_3 \to 0$ in the two-tissue compartment model, $C_T(t) \sim C_R(t)$, which still satisfies convergence.

Demonstration of IMRTM for Simulated Data

Supplemental Figure 13 displays simulated ground truth, low-amplitude release and baseline TACs, along with attempted baseline extractions from MRTM and lp-ntPET. A clear positive bias is observed post-task start with these models.

Supplemental Figure 14 displays the result of IMRTM after 15 iterations; deviations from ground truth are likely due to the simulated data coming from a two-tissue compartmental model (ntPET)[1] while MRTM assumes simplified compartmental modeling.

Supplemental Figure 15 displays the results of IMRTM in the presence of noise, after denoising with IHYPR4D.

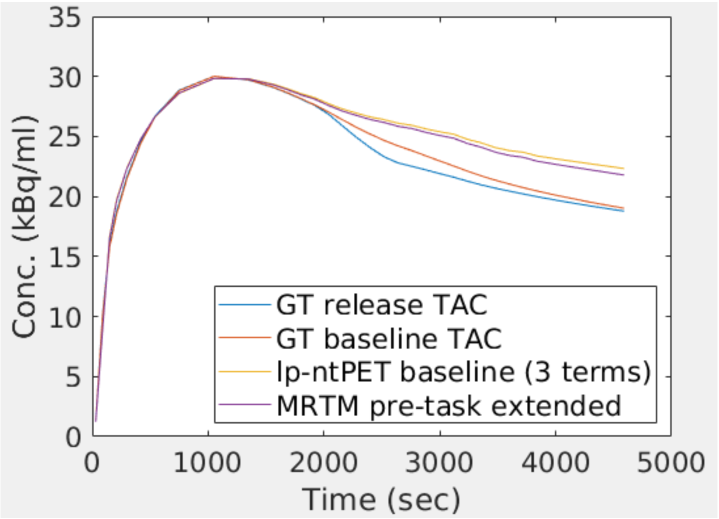

Supplemental Figure 13. Simulated ground truth (GT) baseline TAC, release TAC, and attempted baseline extraction methods using pre-task MRTM and lp-ntPET's first three terms (i.e. setting $\gamma = 0$ following lp-ntPET fitting).

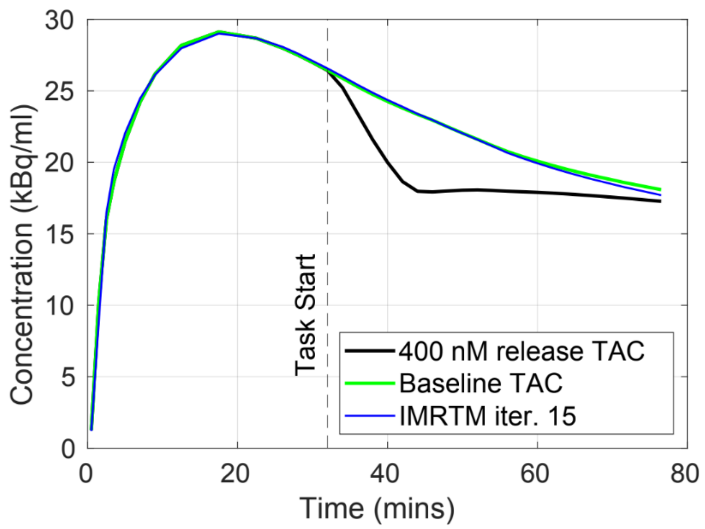

Supplemental Figure 14. Result of IMRTM after 15 iterations, $C_B^{(15)}(t)$, compared to the ground truth baseline TAC $C_B(t)$. Release voxel TAC $C_T(t)$ shown for reference.

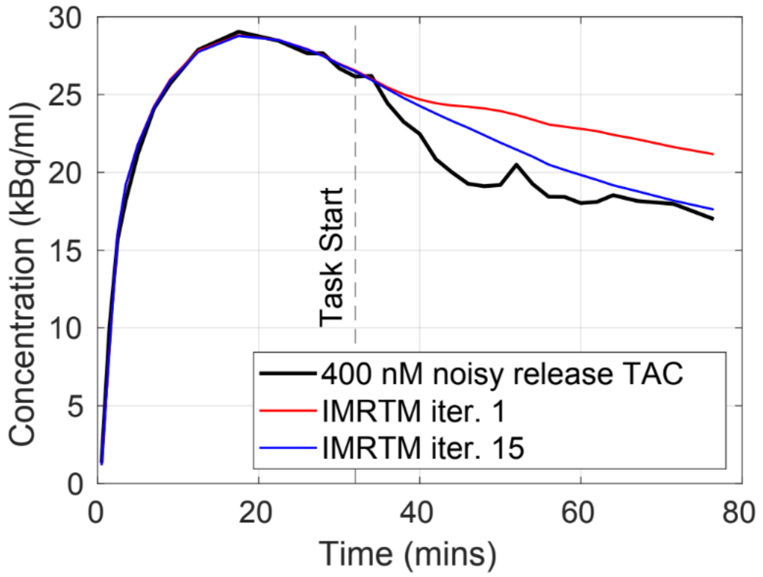

Supplemental Figure 15. Result of IMRTM after 1 and 15 iterations for a noisy simulated high release voxel TAC $C_T(t)$.

Comparison to different baseline methods in RSD

Here we apply RSD with different baseline prediction methods to compare histograms of β values in the presence of noise (Supplemental Figure 16). These baseline prediction methods are (1) applying SRTM2[13] on pre-task TACs and extrapolating the resulting curves, (2) applying IMRTM, (3) applying RSD with replacement by lp-ntPET-derived baseline TACs (RSD-Hybrid-lp-ntPET, i.e. first 3 terms of lp-ntPET), and (4) applying RSD with replacement by IMRTM-derived baseline TACs (RSD-Hybrid-IMRTM, main text). Using the global striatal release simulations (see main text), an ideal histogram would be a small distribution around zero for the release-free right caudate, and a larger distribution at some positive β for the remaining regions.

RSD-Hybrid-IMRTM and IMRTM on its own yield the expected distributions. SRTM2 also has distributions centered where would be expected, but there is a considerable increase in variance. RSD-Hybrid-lp-ntPET uses biased TACs as replacement (see Supplemental Figure 10) and the full distribution is positively biased.

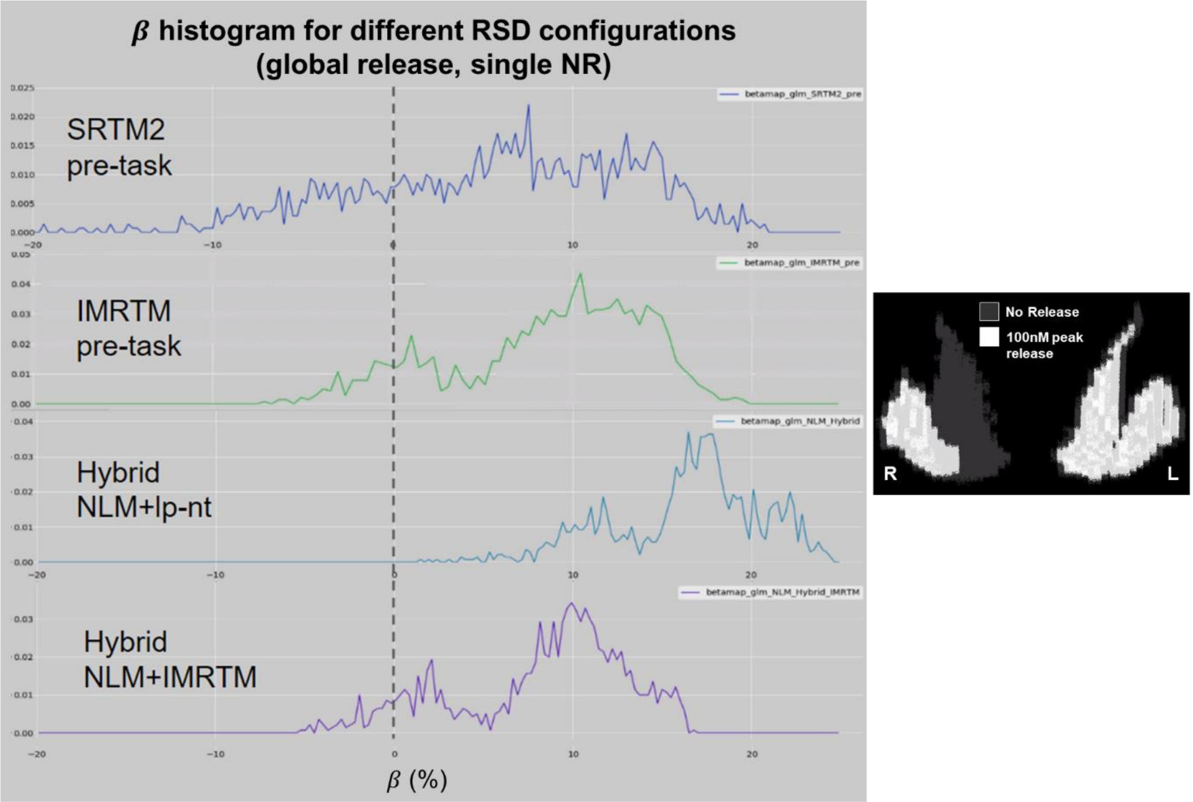

Supplemental Figure 16. Histograms of β estimates from RSD for a variety of baseline prediction methods. The method used in the main text is shown as the final row, RSD-Hybrid-IMRTM.

References

1. Calakos KC, Liu H, Lu Y, et al. Assessment of transient dopamine responses to smoked cannabis. *Drug Alcohol Depend*. 2021;227:108920.

2. Cosgrove KP, Wang S, Kim SJ, et al. Sex Differences in the Brain's Dopamine Signature of Cigarette Smoking. *J Neurosci*. 2014;34(50):16851-16855.

3. Fischl B. FreeSurfer. *Neuroimage*. 2012;62(2):774-781.

4. Morris ED, Yoder KK, Wang C, et al. ntPET: A New Application of PET Imaging for Characterizing the Kinetics of Endogenous Neurotransmitter Release. *Mol Imaging*. 2005;4(4):7290.2005.05130.

5. Wang S, Kim S, Cosgrove KP, Morris ED. A framework for designing dynamic lp-ntPET studies to maximize the sensitivity to transient neurotransmitter responses to drugs: application to dopamine and smoking. *Neuroimage*. 2017;146:701-714.

6. Fisher RE, Morris ED, Alpert NM, Fischman AJ. In vivo imaging of neuromodulatory synaptic transmission using PET: A review of relevant neurophysiology. *Human Brain Mapping*. 1995;3(1):24-34.

7. Pappata S, Dehaene S, Poline JB, et al. In vivo detection of striatal dopamine release during reward: a PET study with [(11)C]raclopride and a single dynamic scan approach. *Neuroimage*. 2002;16(4):1015-1027.

8. Grant AM, Deller TW, Khalighi MM, Maramraju SH, Delso G, Levin CS. NEMA NU 2-2012 performance studies for the SiPM-based ToF-PET component of the GE SIGNA PET/MR system. *Med Phys*. 2016;43(5):2334.

9. Bevington CWJ, Cheng JC, Sossi V. A 4-D Iterative HYPR Denoising Operator Improves PET Image Quality. *IEEE Transactions on Radiation and Plasma Medical Sciences*. 2022;6(6):641-655.

10. Cheng JCK, Bevington CWJ, Sossi V. HYPR4D kernel method on TOF PET data with validations including image-derived input function. *EJNMMI Physics*. 2022;9(1):78.

11. Wang S, Kim S, Cosgrove KP, Morris ED. A framework for designing dynamic lp-ntPET studies to maximize the sensitivity to transient neurotransmitter responses to drugs: Application to dopamine and smoking. *Neuroimage*. 2017;146:701-714.

12. Lippert RN, Cremer AL, Edwin Thanarajah S, et al. Time-dependent assessment of stimulus-evoked regional dopamine release. *Nat Commun*. 2019;10(1):336.

13. Wu Y, Carson RE. Noise reduction in the simplified reference tissue model for neuroreceptor functional imaging. *Journal of cerebral blood flow and metabolism : official journal of the International Society of Cerebral Blood Flow and Metabolism*. 2002;22(12).
